# PKCη/Rdx-driven Phosphorylation of PDK1: A Novel Mechanism Promoting Cancer Cell Survival and Permissiveness for Parvovirus-induced Lysis

**DOI:** 10.1371/journal.ppat.1004703

**Published:** 2015-03-05

**Authors:** Séverine Bär, Jean Rommelaere, Jürg P. F. Nüesch

**Affiliations:** Infection and Cancer Program, Tumor Virology Division (F010), German Cancer Research Center (DKFZ), Heidelberg, Germany; King's College London School of Medicine, UNITED KINGDOM

## Abstract

The intrinsic oncotropism and oncosuppressive activities of rodent protoparvoviruses (PVs) are opening new prospects for cancer virotherapy. Virus propagation, cytolytic activity, and spread are tightly connected to activation of the PDK1 signaling cascade, which delays stress-induced cell death and sustains functioning of the parvoviral protein NS1 through PKC(η)-driven modifications. Here we reveal a new PV-induced intracellular loop-back mechanism whereby PKCη/Rdx phosphorylates mouse PDK1:S138 and activates it independently of PI3-kinase signaling. The corresponding human PDK1phosphoS135 appears as a hallmark of highly aggressive brain tumors and may contribute to the very effective targeting of human gliomas by H-1PV. Strikingly, although H-1PV does not trigger PDK1 activation in normal human cells, such cells show enhanced viral DNA amplification and NS1-induced death upon expression of a constitutively active PDK1 mimicking PDK1phosphoS135. This modification thus appears as a marker of human glioma malignant progression and sensitivity to H-1PV-induced tumor cell killing.

## Author Summary

The H-1 protoparvovirus (H-1PV) is the first replication-competent member of the Parvoviridae family to undergo a phase I/IIa clinical trial in patients suffering from glioblastoma multiforme. Although the intrinsic oncotropism and oncolytic activity of protoparvoviruses are well known, the underlying molecular mechanisms remain elusive. Here we identify a PV-induced intracellular loop-back mechanism that promotes PV replication and cytotoxicity through PI3-kinase-independent stimulation of PDK1 and of the PKC and PKB/Akt1 downstream kinases. This mechanism involves PKCη/Rdx-mediated phosphorylation of PDK1 (at S138 in mouse or S135 in human). Interestingly, this phosphorylation appears as a hallmark of highly aggressive brain tumors. Although H-1PV does not promote it in normal human cells, experimentally administered activated PDK1 variants were able to sensitize these cells to virus infection. These data lead us to propose PDK1phosphoS135 as a new candidate marker for monitoring tumor progression and responsiveness to oncolytic parvovirotherapy, particularly in the case of highly aggressive brain tumors. Furthermore, the sensitivity of PDK1phosphoS135-positive cell lines to inhibitors of PKCη/Rdx argues for considering this complex as a potential target for anticancer drug development.

## Introduction

Protoparvoviruses (PVs) are non-enveloped icosahedral particles 24 nm in diameter, with a 5.1 kb linear single-stranded DNA genome encoding two capsid (VP) and several nonstructural (NS) proteins. Many rodent PVs, including H-1PV, were initially discovered as opportunistic infectants of human-cancer-derived cell lines [1] and are now widely recognized for their intrinsic oncotropism and oncolytic activity. This, together with their non-association with human disease, has led to a first phase I/IIa clinical trial of wild-type replication-competent H-1PV in glioma patients [2]. NS1, the major protoparvoviral regulatory protein, is required for multiple steps in the virus life cycle, ranging from viral DNA amplification and *trans*-regulation of viral and cellular transcription to the egress and spread of progeny particles [3]. Because it interferes with multiple cellular pathways, NS1 appears as the main cytotoxic agent responsible for the oncolytic activity of PVs [4,5]. NS1 functioning is tightly regulated by phosphorylation, catalyzed by two kinases: PKCλ and the short-lived PKCη [6,7], both of which require activation by the phosphoinositide-dependent kinase 1 (PDK1). To ensure virus propagation and spread, the PV minute virus of mice (MVM) has evolved a mechanism for stimulating PDK1 and the downstream kinase PKCη in permissive host cells. This activation of PDK1 is associated with its PV-induced *trans-*phosphorylation by (an) unidentified kinase(s) [8].

The PI_3_K (phosphoinositide 3-kinase)/PDK1/protein kinase B (PKB/Akt) signaling cascade regulates pathways involved in the translational control of protein synthesis and in cell metabolism, differentiation, death, and survival [9]. Accordingly, human cancers frequently display somatic mutations affecting PI_3_K /PDK1/PKB signaling. The master kinase PDK1 has multiple downstream targets besides PKB/Akt1, including SGK, S6K1, RSK, PKN, and the PV-regulating PKCs [10]. PDK1 exerts constitutive basal activity, but it is strongly upregulated by PI(3,4,5)P_3_, produced by PI_3_K, [11,12], itself controlled by growth factor receptor signaling [13]. Additional upregulations include src-family-kinase-driven tyrosine phosphorylations, which act in cooperation with the chaperone Hsp90 to stabilize active PDK1 [14].

The PKC protein kinase family comprises three groups (**a**typical, **n**ovel, and **c**lassical or **a, n,** and **c** PKCs) having different regulatory domains and cofactor requirements [15]. These proteins are involved in regulating processes as diverse as cell metabolism, polarity, differentiation, proliferation, motility, survival, and death. In keeping with their functions and their stimulation by phorbol esters, the n and c PKCs have been implicated in cancer progression, but a negative influence on tumorigenesis has also been evidenced. This is best-studied of the widely expressed nPKCs PKCε and PKCδ, which respectively promote cell survival and death [16]. To perform their multiple and very distinct functions, PKCs are tightly regulated. Besides cofactor binding, a series of phosphorylation events, driven by PDK1 and other kinases, trigger conformational changes controlling the activity of PKCs and their interactions with potential substrates [17,18]. For example, nPKCη must first be phosphorylated by PKCλ at its PDK1-docking site before getting activated by PDK1 [8]. In contrast, aPKCλ requires no such priming because, instead of the serine present in nPKCη, it has a glutamic acid creating a constitutively active PDK1-docking site [17]. PKC down regulation occurs through dephosphorylation followed by ubiquitin-associated degradation [17,18]. Additional regulation can be achieved through interaction with adaptor proteins [19].

Upon activation, PKCs undergo a switch from affinity for scaffold structures to association with membranes, mediated by acid lipids and diacylglycerols. ERM-family proteins (ezrin [Ez], radixin [Rdx], moesin [Moe]) act as intermediates between F-actin and membranes [20]. This adaptor function and the involvement of ERM proteins in the same processes as PKCs [21] suggest that the former may act as PKC-regulating auxiliary proteins. This is substantiated by a recent analysis of parvovirus-host cell interactions [22], which demonstrated strong colocalization of PKCη with the ERM-family protein radixin upon PV infection, accompanied by PKCη activation and modulation of PKCη-induced phosphorylation of viral proteins.

The present study aimed to characterize the mechanism underlying activation of PDK1signaling in PV-infected permissive cells (A9 mouse fibroblasts). Our work has led to the discovery of a PV-induced loop-back mechanism where PKCη, in a complex with radixin, phosphorylates PDK1 at S138. Investigation of the corresponding mechanism (PKCη/Rdx-induced phosphorylation of PDK1 at S135) in human cells and glioma samples has led us to propose PDK1phosphoS135 as a marker of both tumor progression and responsiveness to parvovirus treatment and this pathway, as a potential new target for cancer therapy.

## Results

### PV-induced activation of PDK1 in permissive cells

**Fig 1:**
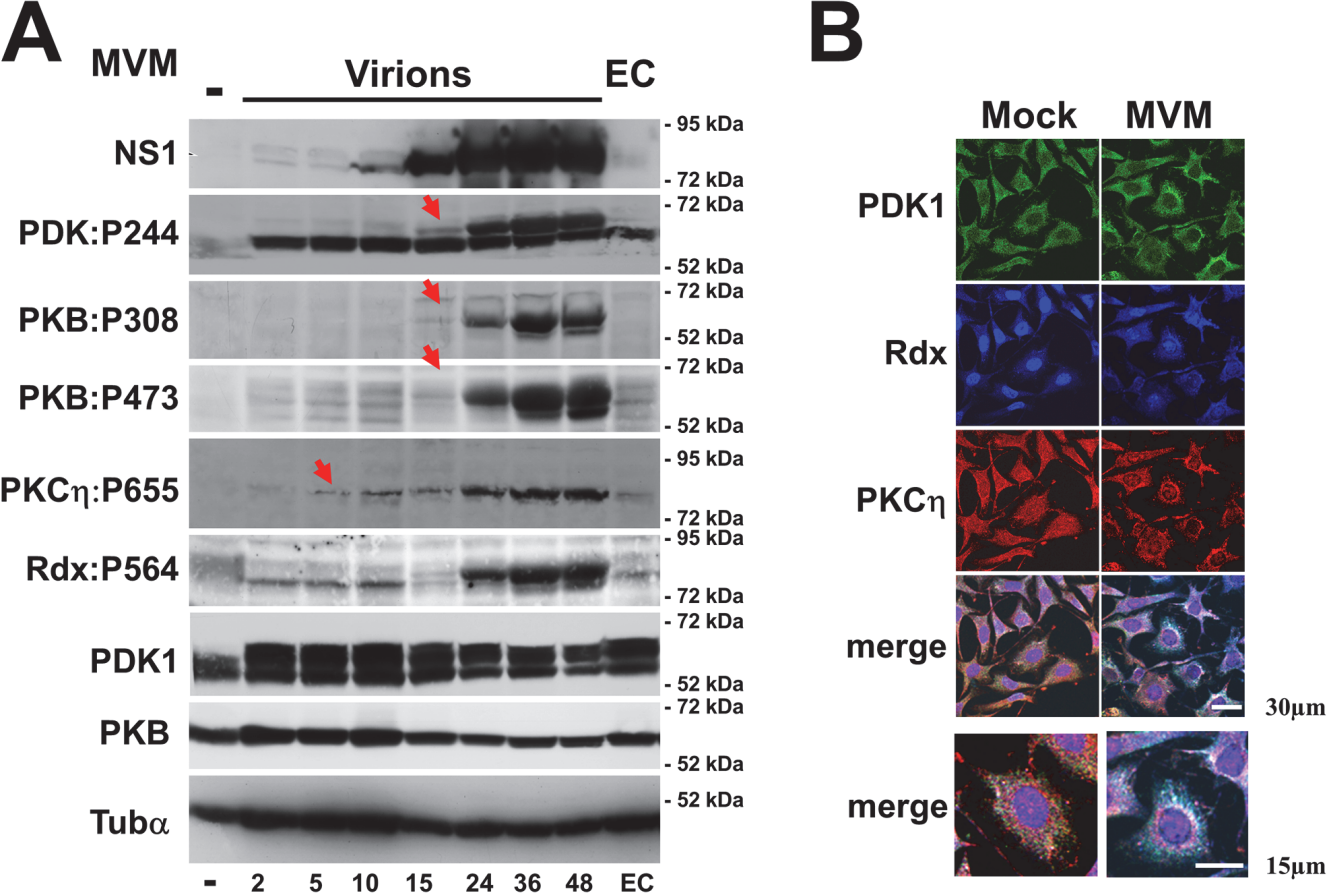
MVM-induced activation of the PDK1/PKC/PKB signaling cascade. As shown previously, MVM activates Rdx [22], PDK1, and PKCη [8] in permissive A9 mouse fibroblasts. This activation is accompanied by PDK1 and PKCη translocation from the plasma membrane to the perinuclear area, where PKCη co-localizes with Rdx [8,22]. (A) Asynchronously growing A9 cells were infected (or not) with CsCl-purified full MVM capsids (30 pfu/cell) or an equivalent amount of empty capsids (EC). Total cell extracts were prepared at the indicated times p.i. Activation of selected cell proteins by MVM was monitored by western blotting on the basis of the proteins’ capacity for auto-phosphorylation (PDK1phosphoS244; PKCηphosphoT655) or of their *trans* phosphorylation at residues known to be essential for activation (Rdx:phosphoT564, PKBphosphoT308 [PDK1 target] and PKBphosphoS473). These protein modifications were detected with phospho-specific antisera. Total amounts of PDK1 and PKB were determined in parallel, and α-tubulin was used as an internal control. It is noteworthy that PKCη activation (starting at 5 h p.i., red arrow) precedes activation of the slower migrating PDK1 form and PKB (starting at 15 h p.i., red arrows). In addition, like PDK1, a slower migrating form of Rdx becomes activated at 15 h p.i. coinciding with its phosphorylation by the NS1/CKIIα complex [22]. (B) Impact of MVM infection on the subcellular distribution of PDK1, PKCη, and radixin. A9 cells grown on spot slides were infected (or not) with CsCl-purified MVM (30 pfu/cell) and examined 36 h p.i. by confocal laser scanning microscopy to confirm colocalization of PDK1 (green), PKCη (red), and Rdx (blue). Colocalization appears white in the merge and was quantified with Image J software. Scale bars: 30 and 15 μm, as indicated.

MVM-infected A9 cells display changes in PDK1/PKC/PKB signaling that are essential to promoting a productive infection. This is illustrated by the results presented in Fig. 1, which confirm and extend previous findings. Firstly, MVM infection triggers activation of PDK1 and of the downstream kinases PKCη [8] and PKB/Akt1 (Fig. 1A). This is accompanied by relocation of active PDK1 and PKCη from plasma membrane ruffles to the nuclear periphery [8], where they both co-localize with the ERM-family cellular auxiliary protein radixin (Rdx) ([22] and Fig. 1B), involved in MVM propagation and spreading and known to modulate PKCη-driven phosphorylation of NS1. Surprisingly, activated PKCη was detected after the onset of viral protein synthesis but before activation of PDK1 (Fig. 1A). These observations led us to hypothesize the existence of a loop-back mechanism where Rdx acting as an adaptor protein controls the subcellular localization, activity, and/or substrate specificity of PKCη so as to activate PDK1.

**Fig 2:**
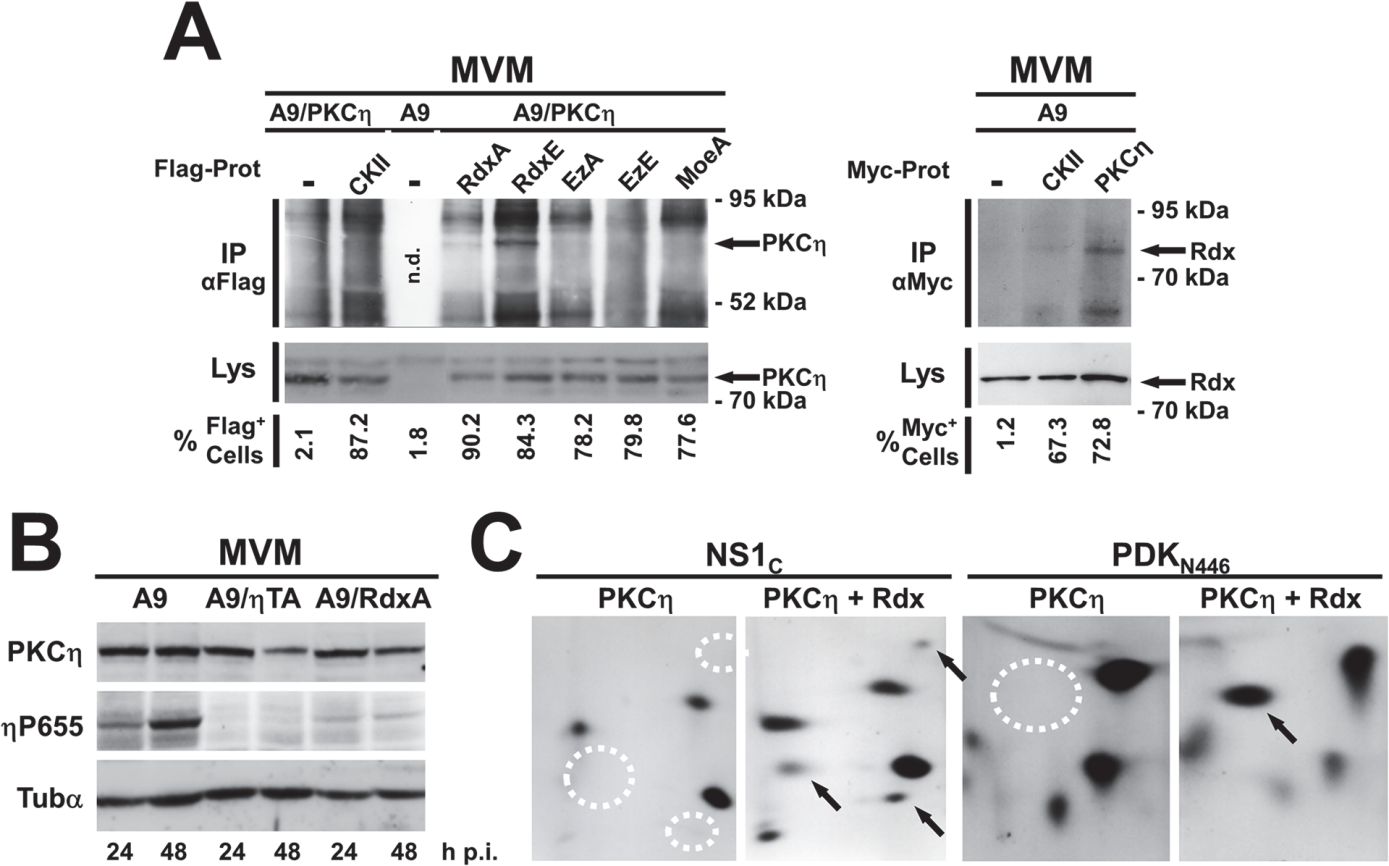
Rdx interacts with PKCη and controls its activity and substrate specificity. (A, B) A9 cells and derivatives expressing the gene encoding the indicated variant protein under the control of the NS1-inducible P38 promoter were infected with MVM (30 pfu/cell) and analyzed at the indicated times p.i. (A) Rdx interacts physically with PKCη inside cells. Left panel: Cell lines expressing MycPKCη (PKCη) alone or together with Flag-tagged CKIIαE81A (CKII), RdxT564A(Rdxa), RdxT564E (RdxE), EzT566A (EzA), EzT566E (EzE), or MoeT547A (MoeA), were harvested 36 h p.i. Co-immunoprecipitation assays were performed under non-denaturing conditions with mouse monoclonal Flag-tag-specific M2 antibodies. Immunoprecipitates (IPαFlag) and, for comparison, whole-cell lysates (Lys) were analyzed by western blotting with rabbit anti-Myc antibodies to detect MycPKCη. The percentage of Flag-positive cells in these lines was determined by immunofluorescence with M2 antibodies (% Flag^+^ cells). Arrows indicate the position of MycPKCη in CoIPs. n.d. stands for “not determined”. Right panel: A9, and cell lines expressing MycPKCη or MycCKIIα were harvested 36 h p.i. Co-immunoprecipitation assays were performed under non-denaturing conditions with anti-Myc antibodies. Immunoprecipitates (IPαMyc) and, for comparison, whole-cell lysates (Lys) were analyzed by western blotting with goat anti-Rdx antibodies to detect endogenous radixin. The percentage of Myc-positive cells in these lines was determined by immunofluorescence with anti-Myc antibodies (% Myc^+^ cells). Arrows indicate the position of Rdx in CoIPs (B) Rdx controls the activity of PKCη in MVM-infected A9 cells. A9 cells and derivatives expressing dominant-negative PKCηT512A (ηTA) or RdxT564A (RdxA) were harvested at the indicated times p.i. and analyzed by western blotting. As a measure of endogenous PKCη activity, the amount of PKCη auto-phosphorylated at T655 (ηP655) was estimated as compared to the total amount of the kinase (PKCη). The loading control was α-tubulin (Tubα). (C) Radixin controls the substrate specificity of PKCη. The MVM NS1 *trans-*activation domain, aa 545–672 (NS1_C_) and C-terminally truncated PDK-1_N446_ were phosphorylated *in vitro* by PKCη alone (PKCη) or with radixin (PKCη/Rdx) and their tryptic phosphopeptides were detected. Peptides labeled specifically in the presence of Rdx are indicated with arrows (presence) or dotted circles (absence).

To test this hypothesis, we first examined whether Rdx or other ERM-family proteins might interact physically with PKCη and modulate its activity. A9 cells and derivatives expressing Myc-tagged PKCη (MycPKCη), either alone or in the presence of a Flag-tagged ERM variant, were infected with MVM and harvested 24 h post-infection. Complexes containing Flag-tagged ERM were recovered by immunoprecipitation with anti-Flag and tested for the presence of MycPKCη by western blotting with anti-Myc. As shown in Fig. 2A (left panel), MycPKCη was pulled down with both active RdxE (RdxT564E) and, to a minor extent, inactive RdxA (RdxT564A). No MycPKCη was detected in the absence of recombinant Flag-ERM or in the presence of Flag-Ez or Flag-Moe. The specificity of the interaction was confirmed with the reverse co-immunoprecipitation assay with αMyc (Fig. 2A right panel). While immunoprecipitation with MycPKCη was able to capture significant amounts of endogenous Rdx, only minor quantities were detected in absence of Myc-tagged proteins or MycCKIIα. PKCη thus appears to bind specifically to Rdx in MVM-infected A9 cells. We next tested how this binding might affect the properties of PKCη. First, MVM-infected A9 cells and derivatives expressing dominant-negative RdxA were harvested 24 and 48 hours post-infection and autophosphorylation of endogenous PKCη at T655 was measured by western blotting with an antibody against PKCη:phosphoT655 (Fig. 2B). A cell line expressing dominant-negative PKCη (ηTA: PKCηT512A) served as control. Both the control cells and the RdxA-expressing cells showed a strongly reduced level of PKCη:phosphoT655, indicating that the Rdx-PKCη interaction controls the activity of PKCη. Next, to see if Rdx binding to PKCη might influence the substrate specificity of the kinase, we performed *in vitro* phosphorylation assays followed by tryptic phosphopeptide profiling. For this, a purified non-phosphorylated recombinant peptide, either PDK1_N446_ (aa 1–446) or NS1_C_ (aa 545–672) used as control, was incubated with PKCη and γ^32^P-ATP in the presence or absence of purified functionally active Rdx (Fig. 2C). Whichever fragment was used, some ^32^P-labeled peptides appeared only when Rdx was included in the reaction. Taken together, these results suggest that Rdx acts as an adaptor to control PKCη activity and substrate specificity and further support our hypothesis that in the perinuclear area, a PKCη/Rdx complex mediates PDK1 phosphorylation and upregulation.

**Fig 3:**
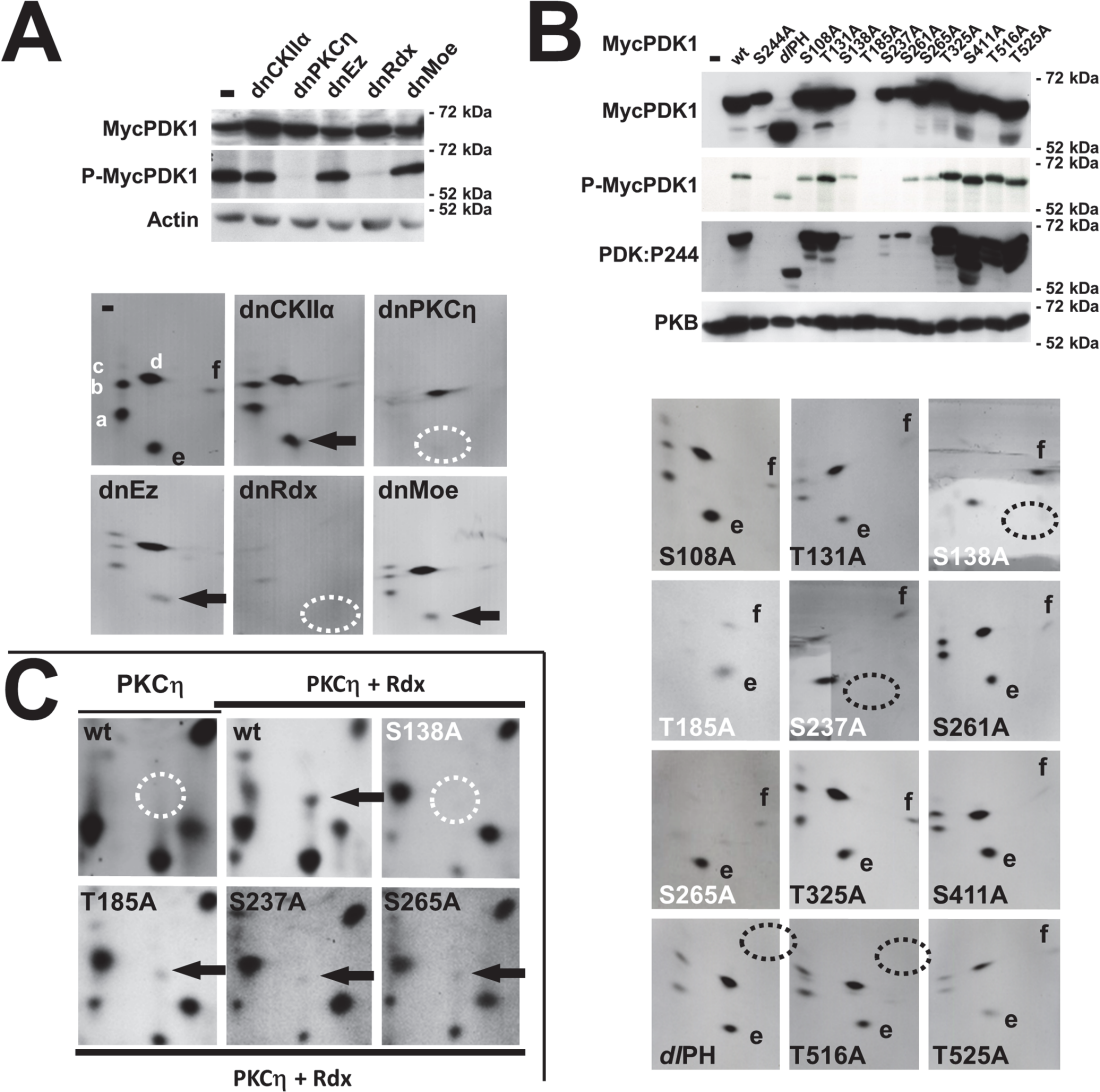
MVM induces PDK1 activation through PKCη/Rdx-driven *trans*-phosphorylation at S138. (A, B) A9 cell derivatives expressing MycPDK1_X_ alone or in the presence of the indicated dominant-negative effector protein were infected with MVM (30 pfu/cell) and, when indicated, metabolically labeled 24 h p.i. and further processed for analysis of the PDK1 tryptic phosphopeptide pattern. (A) PKCη/Rdx causes the appearance of a specific PDK1 phosphopeptide and controls the overall phosphorylation of PDK1. Top: Phosphorylated MycPDK1 was determined by metabolic ^32^P-labeling of MycPDK1 after recovery by immunoprecipitation (P-MycPDK1). The total amount of MycPDK1 (MycPDK1) was measured 24 h p.i. by western blotting. Actin was used as a loading control. *Bottom*: As shown by Lachmann and coworkers, the tryptic phosphopeptide pattern of metabolically labeled MycPDK1 comprises peptides (a-d), which are autophosphorylated by PDK1 and absent in catalytically inactive PDK1S244A. In contrast, peptides (e, f), which are present in the profile of inactive PDK1S244A are targeted by (an)other kinase(s) [8]. Phosphorylation of “e” (arrow) was found to depend on both PKCη and Rdx (absence = dotted circles). dnCKII: CKIIαE81A, dnPKCη: PKCηT512A, dnEz: EzT566A, dnRdx: Rdx*dl*[P], dnMoe: MoeT547A). (B) Phosphorylation and activity of PDK1 mutants. Top: Overall phosphorylation of MycPDK1 measured after metabolic labeling 24 h p.i. (P-MycPDK1). Total (MycPDK1) and functionally active, autophosphorylated PDK1phosphoS244 (PDK1:P244) were quantified by western blotting. PKB was used as an internal loading control. Bottom: Tryptic phosphopeptide patterns of inactive (white) and active (black) PDK1 mutants. Specific loss of *trans-*phosphorylated peptides “e” (produced by PKCη/Rdx) and “f” is indicated by dotted circles. (C) PKCη/Rdx phosphorylates PDK1:S138 in vitro. PDK1_N446_ was phosphorylated *in vitro* in the presence of PKCη alone (PKCη) or PKCη with radixin (PKCη/Rdx), and its tryptic phosphopeptide profile obtained. Phosphopeptides found specifically in the presence of Rdx are indicated with arrows (presence) or dotted circles (absence).

To further test our hypothesis, we measured the activity and phosphorylation of (recombinant) PDK1 in MVM-infected A9 cells where either PKCη, another candidate protein kinase, or an ERM-family protein was inactivated by expression of a dominant-negative mutant (Fig. 3A). As measured by metabolic ^32^P-labeling, the steady-state level of (Myc)PDK1 phosphorylation was found to be markedly reduced in cells expressing either dnPKCηT512A or dnRdx*dl*[P], as compared to mock-, dnCKIIα-, dnEz-,and dnMoe-expressing cells (Fig. 3A top panel). Endogenous PDK1 activity showed similar modulation (S1 Fig.), suggesting that PKCη/Rdx controls PDK1 activity in MVM-infected A9 cells. To distinguish PKCη/Rdx-mediated phosphorylation from autophosphorylation, tryptic phosphopeptide analyses were performed (Fig. 3A bottom panel). The wild-type pattern consists of six phosphopeptides (a-f), four of which (a-d) are dependent on PDK1 kinase activity (i.e. absent when catalytically inactive PDK1:S244 is used), while other kinases appear to be responsible for the other two (e, f) [8]. Although the overall phosphorylation of MycPDK1 was markedly reduced upon inactivation of PKCη or Rdx, a faint, incomplete autophosphorylation pattern (peptides a-d) remained visible allowing the identification/localization of the individual spots. Interestingly, besides the pronounced reduction of autophosphorylation (a-d), no labeling of peptide “e” could be detected after expression of PKCηT512A or Rdx*dl*[P]. Considering that the appearance of this phosphopeptide is independent of the intrinsic catalytic activity of PDK1 (i.e. present in PDK:S244A [8] and S265A [Fig. 3B bottom panel]) and since no differences in accumulation of MycPDK1 occurs under these conditions (Fig. 3A top panel), this observation strongly suggests that *trans*-phosphorylation of PDK1on peptide “e” is mediated by PKCη/Rdx and controls the overall activity of PDK1. As inhibition of CKIIα or of the ERM-family protein ezrin or moesin did not impede PDK1 *trans*-phosphorylation (peptide “e”) or autophosphorylation (peptides a-d), PKCη and Rdx appear as specific regulators of PDK1.

To identify the site(s) of PKCη/Rdx-driven *trans*-phopshorylation in PDK1, candidate target phosphorylation sites were mutated from serine/threonine to inert alanine, and A9 cell lines expressing the Myc-tagged PDK1 variants were generated. As shown in Fig. 3B top panel, some mutations [S108A, T131A, T325A, S411A, T516A, T525A, deletion of the whole PH-domain (*dl*PH)] had little to no effect on PDK1 phosphorylation or activity, while others [S138A, S237A, S261A, S265A] strongly reduced PDK1 activity (PDKphosphoS244) and steady-state phosphorylation (P-MycPDK1). As MycPDK1:T185A was hardly detectable by western blotting, this modification probably affects the stability of the polypeptide. As shown in Fig. 3B bottom panel and in agreement with the results in the top panel, kinase-active mutants yielded clearly detectable auto-phosphorylated peptides (a-d) and *trans-*phosphorylated peptides (e, f), whereas mutants S138A, T185A, S237A, and S265A gave rise to very faint, incomplete patterns. With mutants S138A and S237A, no peptide “e” was observed (dotted circles Fig. 3B bottom panel). This suggests that residues S138 and S237 could be (direct or indirect) targets of the PKCη/Rdx complex, and that, in the absence of phosphorylation at these positions, PDK1 loses its kinase activity. Although no effects on intrinsic enzyme activity were observed after *dl*PH deletion or T516A substitution, these mutations abolished phosphorylation of peptide “f” (dotted circles), suggesting that T516 may be another *trans*-phosphorylation site in PDK1. Interestingly, substitution of glutamic acid for this residue renders PDK1 constitutively active as regards PKB activation [23], and phosphorylation at this site is specifically triggered upon MVM infection [8].

To identify PDK1 phosphorylation sites directly targeted by the PKCη/Rdx complex, we performed *in vitro* tryptic phosphopeptide analyses (Fig. 3C). In agreement with Fig. 2C, a single PDK1 phosphopeptide was specifically induced in the presence of Rdx (arrow vs. dotted circle). This peptide was not visible upon mutation of S138 to alanine, while it was visible in all the other mutants. Together with the above evidence, this result indicates that PKCη/Rdx phosphorylates PDK1 at residue S138, thereby activating the kinase.

### PKCη/Rdx-mediated phosphorylation of PDK1:S135 in human tumor cell lines: impact on cell metabolism and survival

**Fig 4:**
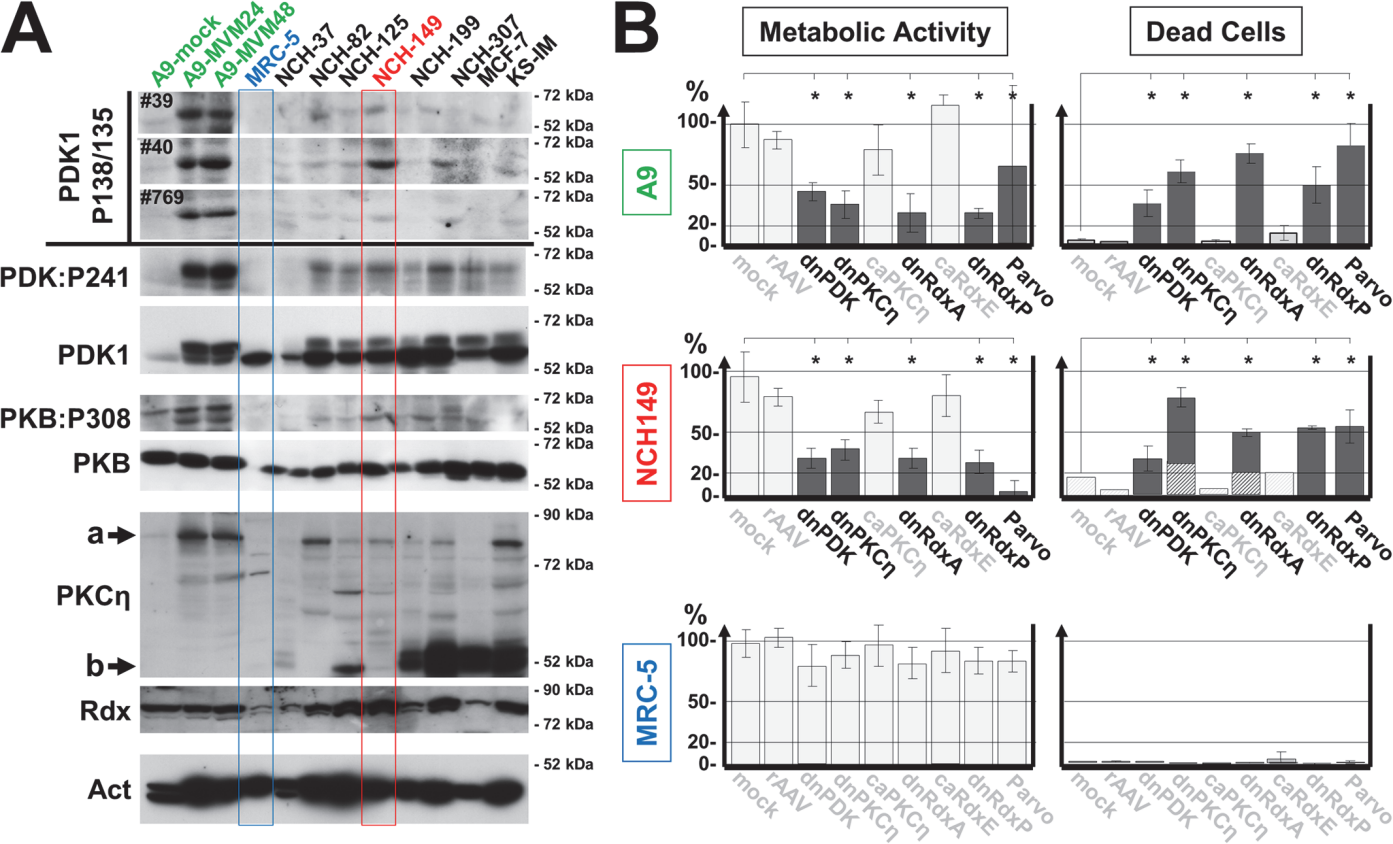
Detection of PDK1phosphoS135 in human cancer-derived cell lines. **(A)** Whole-cell extracts of the indicated human tumor-derived cell lines were analyzed by western blotting. *Top*: PDK1phosphoS135 (human)/phosphoS138 (mouse) was detected with immunoaffinity-purified phosphospecific antisera from three individual immunizations (#39, #40, and #769). Mock- and MVM-infected A9 cells were used as controls of antiserum specificity. *Bottom*: The same extracts were analyzed to determine their PDK1 (PDK1), PDK1phospho308, and total PKB, PKCη, and Rdx contents. α-Tubulin was used as loading control. a, full-length PKCη; b, PKCη proteolytic cleavage product PKMη. The significant level of PKMη seen in some of the examined cancer cell lines could be indicative of upregulated kinase activity, as this fragment can result from cleavage of the regulatory domain and/or increased the turnover of activated protein [40]. (B) Impact of PKCη and Rdx on cell metabolic activity and survival. The indicated cell lines were transduced with a rAAV (10^4^ viral genomes/cell) expressing a dominant-negative (dn) or constitutively active (ca) form of the indicated signaling protein under control of the PV P4 promoter. 72 h post transduction, the cells were labeled for 30 min with Mitotracker and mitochondrial activity was measured by confocal laser scanning microscopy, quantified with Image J software as relative light intensity per cell, and expressed as a percentage of the value obtained for mock-treated cells (light gray columns). In parallel, proportions of dead cells (dark gray columns) and apoptotic cells (hatched columns) were measured, respectively, by PI (necrosis) and DAPI staining (detection of apoptotic bodies). The data presented are means with standard-deviation bars of three individual experiments, each involving > 200 cells per sample. dnPDK, PDK1K204M; dnPKCη, PKCηT512A; caPKCη, PKCηA160E; dnRdxA, RdxT564A; caRdxE, RdxT564E; dnRdxP, Rdx*dl*[P]; For comparison, viability was also measured 24 h after infection of A9 cells with MVM and of NCH149 and MRC-5 cells with H-1PV. Treatments that significantly (p<0,01) impaired cell metabolic activity and/or viability are indicated in black and marked by astericks. rAAV-mediated transduction efficiencies were checked by confocal microscopy (S7 Fig.).

Constitutive activation of the PDK1/PKB signaling cascade is a hallmark of highly invasive cancers, and viruses exploit it to extend the lifespan of infected cells under stress [9,24]. This led us to investigate whether the PV-inducible PKCη/Rdx-mediated phosphorylation of PDK1 at S138 (in mouse) or S135 (in human) might be a cancer pathway leading to constitutive PDK1 activation. Several human cancer cell lines were analyzed for PDK1phosphoS135 and compared with normal diploid fibroblasts. As shown in Fig. 4A, PDK1phosphoS135 was detected (along with Rdx and PKCη) in the Kaposi sarcoma (KS) cell line and all six glioma (NCH) cell lines, but not in normal diploid MRC-5 cells. In most tumor cell lines, phosphorylation of PKB at T308 was also observed, suggesting PDK1:S135 phosphorylation mobilizes an intracellular PI_3_K-independent survival pathway in cancer cells. It should be noted, however, that MCF-7 mammary carcinoma cells, although proficient in PKB:T308 phosphorylation, showed no significant PDK1phosphoS135 signal.

Normally, PDK1 is a master kinase regulating cell metabolism and survival, in conjunction with PKB. Both kinases are tightly controlled by the availability of cofactor PIP_3_, produced by PI_3_K, which in turn is regulated by growth-factor-dependent receptor tyrosine kinases. Stress signaling counteracts this pathway, causing cell death. We hypothesized that PKCη/Rdx-driven phosphorylation of PDK1 might activate the kinase independently of PIP_3_ by altering its conformation. If so, interrupting this loop-back mechanism should impair cell metabolism and cause cell death. We tested this in several cell lines, independently of any PV infection, by transducing them with a rAAV vector overexpressing a mutant form of PDK1, PKCη, or Rdx. The cell lines used were A9 (where we have characterized this pathway after MVM infection), the PDK1phosphoS135-positive, H-1PV-permissive human glioblastoma-derived cancer cell lines NCH149 and NCH82 (Figs. 4A and S2) (known to display enhanced PKB/Akt1 activity and for resistance to apoptosis inducers ([25], and the PDK1phosphoS135-negative, H-1PV-resistant BJ-1 (foreskin) and MRC-5 (embryonic lung) normal human diploid fibroblasts (Figs. 4A and S2), which are fairly insensitive to PDK1 silencing [26]. Metabolic activity (Mitotracker staining) and death by apoptosis/necrosis (nuclear fragmentation/PI-staining) were measured by immunofluorescence staining 48 h post rAAV treatment (S3A Fig.). As summarized in Figs. 4B and S3B, knockdown of endogenous PDK1, PKCη, or Rdx significantly (p<0,01) reduced the metabolic activity of A9, NCH149, and NCH82 cells, causing a large proportion (p<0,01) of the cells to die. No effect was seen with the control vector or in the presence of constitutively active caPKCη or caRdx. Normal human cells showed only minor fluctuations in metabolic activity and no apparent cell death. These data support our hypothesis that PKCη/Rdx-mediated phosphorylation of PDK1 at S135/S138 controls cell metabolic activity and viability of cancer cells.

**Fig 5:**
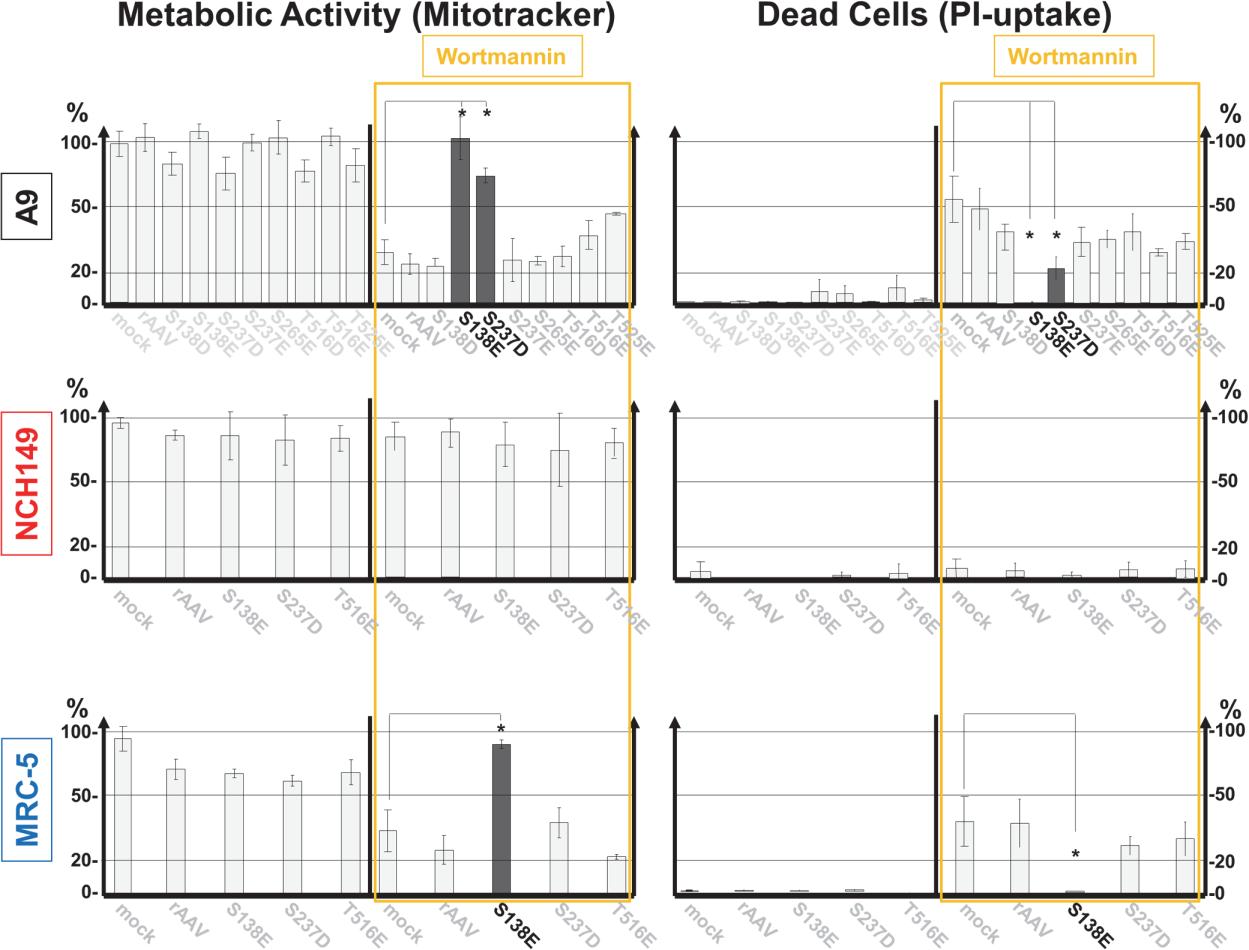
Impact of caPDK1 on the growth factor dependence of cell metabolic activity and survival. Each indicated cell line was transduced with a rAAV (10^4^ rAAV genomes/cell) expressing mutant PDK1 under the control of the PV P4 promoter. 72 h post transduction, the cells were treated (or not) for 4 h with 0.5 μM wortmannin prior to labeling for 30 min with Mitotracker. Mitochondrial activity and cell death were measured as described in the legend of Fig. 4. The constitutively active mutant PDK1:S138E (in A9 cells PDK1:S138E and to a lesser extent PDK1:S237D) significantly (p<0,01) reconstituted metabolic activity and prevented cells from undergoing death through necrosis (indicated by astericks). Thus, PDK1:S138E (and at least in part in A9 PDK1:S237) appeared to render cell viability independent of growth factor signaling via the PI3 kinase (marked black). It should be noted that PDK1:S138E mimics PDK1phophoS135 modification detected in non-transduced NCH149 (Fig. 4A). Transduction efficiencies were checked by confocal microscopy (S7 Fig.).

To further address this issue, we generated constitutively active PDK1 variants mimicking PKCη/Rdx-driven phosphorylation by replacing candidate serine/threonine residues with aspartic or glutamic acid residues. To test the activity of these mutants in A9 cells, we measured PDK1:S244 autophosphorylation and PKB:T308 *trans*-phosphorylation by immunofluorescence microscopy after transfection with plasmid constructs. Strong signals were obtained with PDK1:S138E and PDK1:S237D, suggesting that both mutants have enhanced activity (S4 Fig.). These and other candidates were then transduced by rAAV vectors into A9, NCH149, NCH82, BJ-1, and MRC-5 cells and the impact of their expression on cell metabolic activity and survival was evaluated in the presence and absence of the PI_3_K inhibitor wortmannin (Figs. 5 and S5). A9, BJ-1, and MRC-5 cells treated with this drug showed dramatically reduced cell metabolic activity and massive death when mock-treated or transduced with any rAAV except rAAV:PDK1:S138E and, for A9 cells rAAV:PDK1S237D as well (p<0,01). The cancer cell lines NCH149 and NCH82 resisted wortmannin treatment whether transduced or not. These cells should indeed be at least partly independent of growth factor signaling since, unlike normal cells, they produce PDK1phosphoS135 through PKCη/Rdx-mediated phosphorylation (Fig. 4). A9 cells, although capable of activating PDK1 by PKCη/Rdx-mediated phosphorylation, appear to require acceleration of this loop-back mechanism (e.g. through PV stimulation), in agreement with the low level of PDK1phosphoS138 detected in non-infected A9 cells [[8]; Fig. 4]. Altogether, these results strongly suggest that PKCη/Rdx-induced phosphorylation at PDK1:S135 enables cells to remain viable in the absence of growth factor signaling through PI_3_K.

### Stimulation of the PV life cycle through growth-factor-independent PDK1 activation

**Fig 6:**
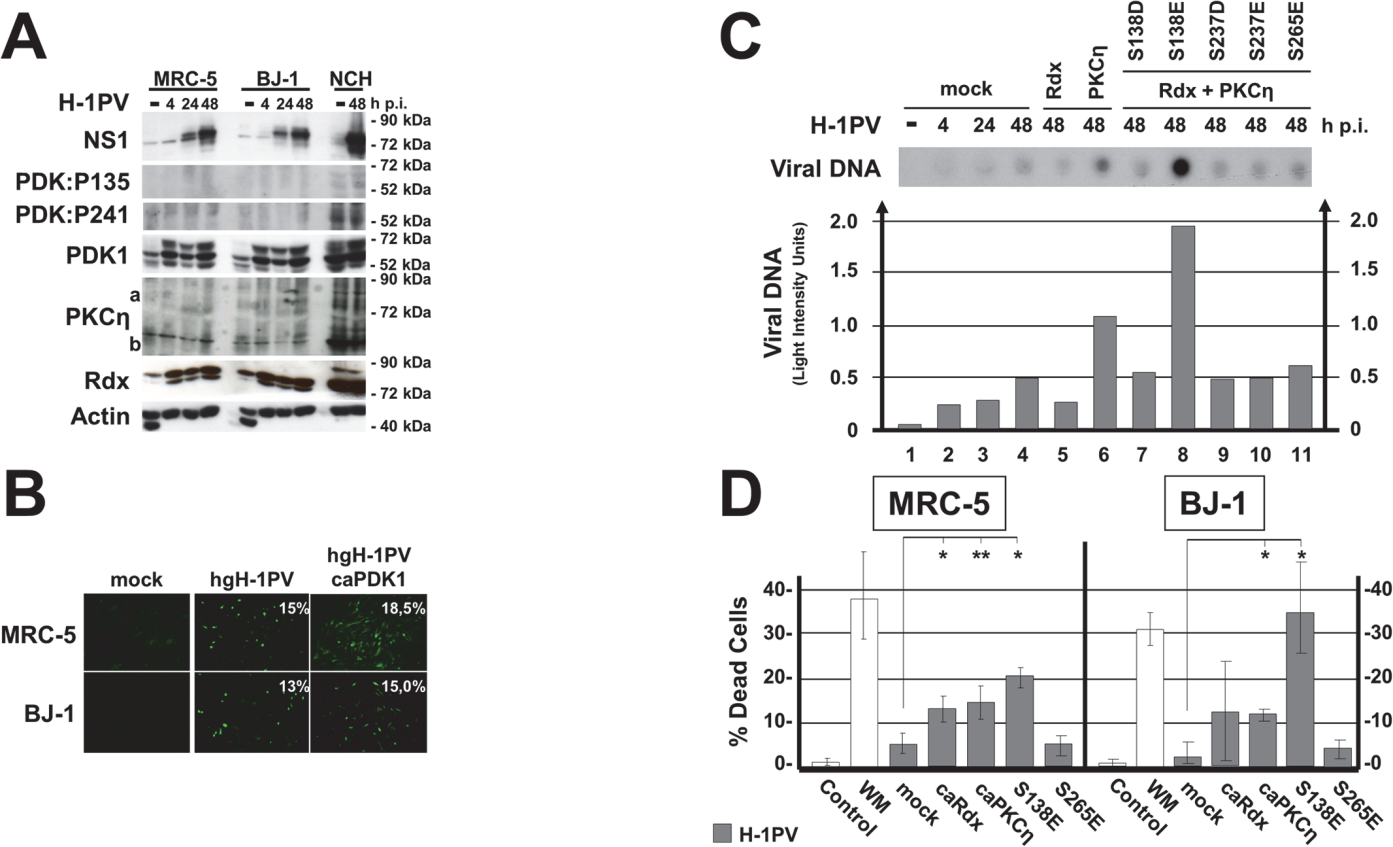
Impact of activated PDK1 on cell permissiveness for PV infection. (A) Normal human fibroblasts (MRC-5 and BJ-1) and human glioma cells (NCH149) were infected (or not) with H-1PV (30 pfu/cell) and harvested at the indicated times p.i. Activated PDK1 was detected by western blotting as PDK1phosphoS135 (PDK:P135) and as autophosphorylated PDK1phosphoS241 (PDK:P241). Total PDK1, viral NS1, PKCη, and Rdx were quantified in parallel. Actin was used as loading control. (B) MRC-5 and BJ-1 cultures grown on spot slides were transfected (or not) with rAAV:PDK1S138E (caPDK1) and infected 24 h thereafter with H-1PV. The proportion of cells having initiated virus replication was determined by measuring NS1 expression by immunofluorescence staining 24 h p.i. (C, D) Normal human cells were transfected (or not) with rAAV (10^4^ rAAV genomes/cell) expressing the indicated PDK1, PKCη, or Rdx variant under the control of the PV P4 promoter and infected with (30 pfu/cell) H-1PV 24 h after transduction. Accumulation of viral DNA in the transfected MRC-5 cells was determined by dot blot hybridization (C, upper panel) and expressed in light intensity units (C, lower panel). (D) The proportion of dead cells was measured by PI staining 48 h p.i. Data are presented as means with standard deviation bars of three independent experiments and significance of the changes compared to mock treated cells were determined by student’s test at p-values p<0,01 (*) and p<0,02 (**), respectively.

Parvovirus propagation depends strongly on active PDK1 and PKCs. In natural hosts, accordingly, this signaling cascade is stimulated after the onset of viral protein synthesis. This led us to hypothesize that PV propagation in illegitimate hosts might depend on constitutively active PDK1/PKC/PKB signaling, since some PV-permissive human cancer cells (e.g. NCH149) display, irrespectively of infection, significantly higher levels of PDK1phosphoS241 and PKBphosphoT308 than normal cells, together with PKCη/Rdx-induced phosphorylation of PDK1:S135 (Fig. 4). We thus wondered if activating PDK1/PKC signaling artificially might sensitize H-1PV-resistant, PDK1phosphoS135-negative normal human cells to H-1PV. To test this we used MRC-5 and BJ-1 cells, which, although unable to support NS1-dependent viral DNA amplification (S2A Fig.), do permit H-1PV entry and the early steps leading to NS1 synthesis (Figs. 6A and S2B). Accordingly, the proportion of cells having initiated PV replication, as determined by NS1 immunostaining, was only slightly higher 24 h post-infection among caPDK1:S138E-treated vs. untreated MRC-5 and BJ-1 cells (Fig. 6B). To activate PDK1/PKC signaling in MRC-5 cells, genes encoding constitutively active PDK1, PKCη, and Rdx variants were transduced into these cells on rAAV vectors and overexpressed prior to infection with H-1PV (Fig. 6C). The reasons for including caPKCη and caRdx in addition to caPDK1 are that PKCη, poorly expressed in MRC-5 cells, is required to activate the viral-DNA-amplification function of NS1 [6] and that Rdx and PKCη act together to activate PDK1. Under these conditions, overexpression of caPDK:S138E was found to stimulate viral DNA amplification significantly (Fig. 6C). Furthermore, as shown in Fig. 6D, treatment with caPDK:S138E strongly sensitized both MRC-5 and BJ-1 cells to H-1PV-induced cell killing, causing a 5- to 10-fold increase in the proportion of dead cells after PV infection (p<0,01). In agreement with their action upstream from PDK1, individually overexpressed caPKCη (and for MRC-5 caRdx as well) had a significant (p<0.02), but less pronounced PV-sensitizing effect on normal human fibroblasts, too. The PDK1/PKC signaling cascade thus appears important for PV oncotropism, since its forced activation in normal human cells confers some degree of permissiveness for PV replication and cytotoxicity.

### Occurrence of PDK1:S135 phosphorylation in human cancer tissues

**Fig 7:**
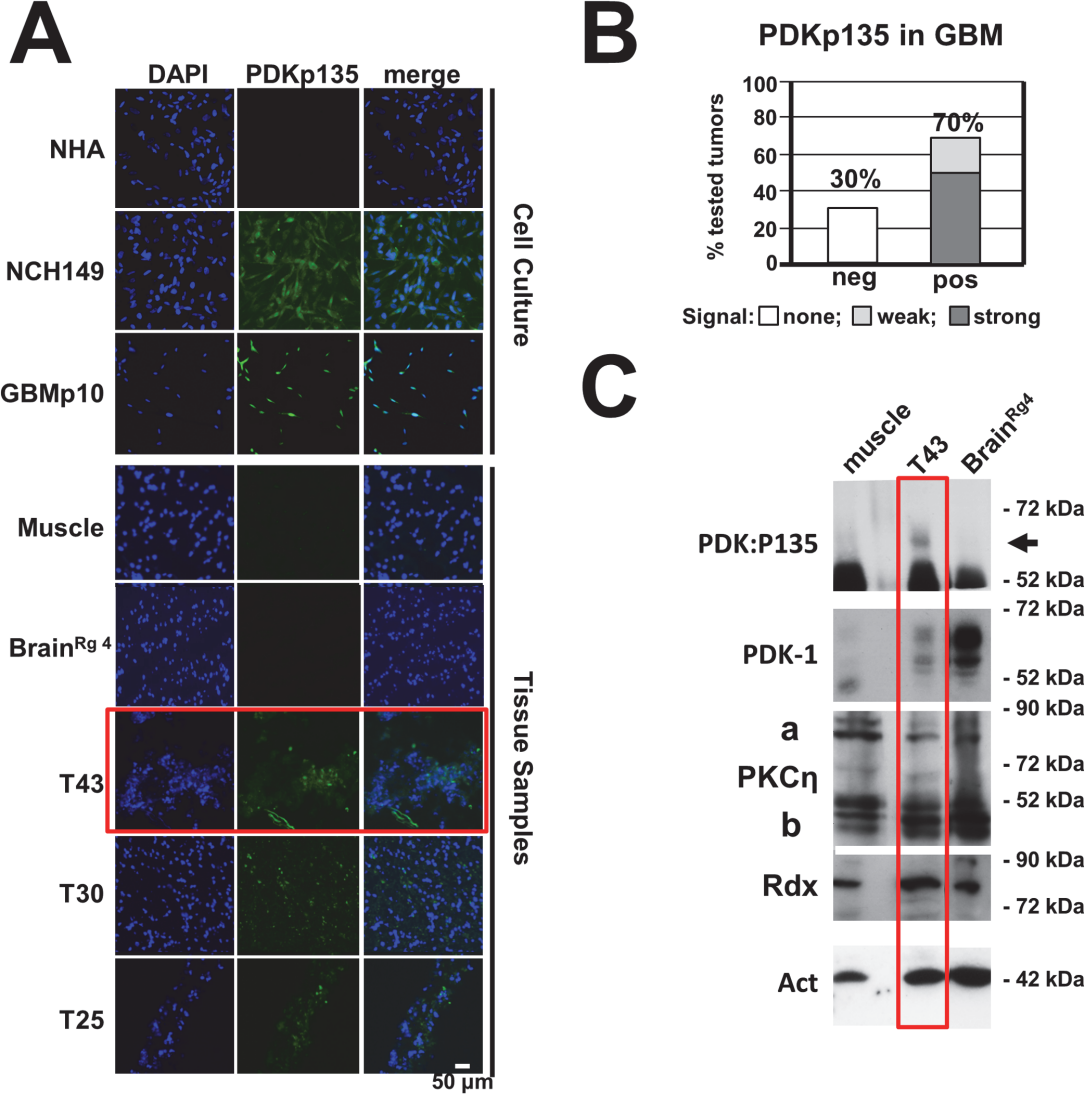
Detection of PDK1phosphoS135 in human cancer tissues. (A) Cryosections of human brain tumors were analyzed for the presence of PDK1phosphoS135 by immunostaining with specific monoclonal antibodies (PDKp135) and counterstaining with DAPI. Normal human astrocytes, muscle tissue samples and a safety margin of healthy looking brain tissue of tumor #56 were used as negative controls while two glioma-derived cell lines (NCH149, GBM21) served as positive controls. Scale bar, 50 μm. (B) Quantitation of the IF microscopy data (see S6 Fig.) shows that 70% of the analyzed tumor samples (n = 36) contained PDK1phosphoS135-positive cells, 50% with a strong and 20% with a weak signal. (C) The presence of PDK1phosphoS135 in tumor T43 was confirmed by western blot analysis of whole-cell extracts with immunoaffinity-purified phosphospecific antiserum (Rabbit #769). Normal muscle tissue and Brain^Rg4^ were analyzed in parallel for comparison. Total amounts of PDK1, PKCη/Rdx, and actin were also determined.

As aberrant activation of PDK1 is thought to contribute to cancer progression and tumor cell invasiveness [9,27] and as PDK1phosphoS135 is detected in tumor cell lines, we performed immunofluorescence staining to detect this modification on cryosections of tumors resected from patients suffering from glioblastoma multiforme, a highly invasive brain cancer. As shown in Figs. 7A, 7B, and S6, about 70% of the examined tumor samples (n = 36) tested positive for PDK1phosphoS135, while cultured normal human astrocytes, normal muscle tissues and a sample of safety margin of healthy looking tissue of tumor #56 (Brain^Rg4^) were negative. These results were confirmed by western blotting, as illustrated in Fig. 7C for specimen #43: PDK1phosphoS135 was found in the brain tumor sample together with PKCη and Rdx, but not in muscle and “normal” brain tissue. These results suggest that intracellular PKCη/Rdx-driven activation of PDK1 through phosphorylation at residue S135 takes place in a large proportion of human gliomas, where it may contribute to cancer progression.

## Discussion

**Fig 8:**
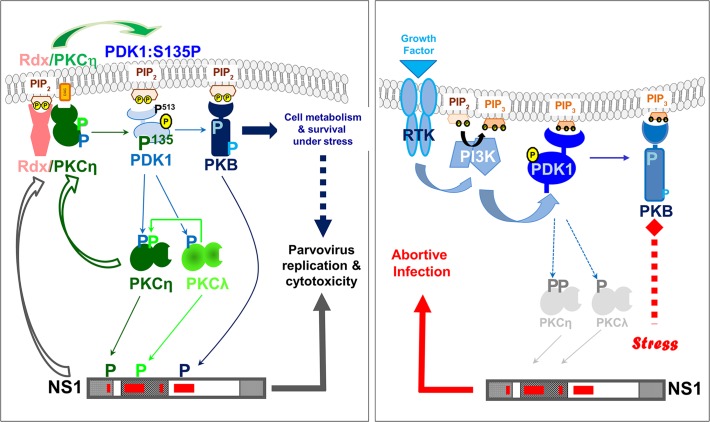
Interdependence of PV propagation and activation of the PDK/PKC/PKB signaling cascade. Schematic representation of parvoviral interaction with the PDK1/PKC/PKB signaling cascade in permissive host and human cancer cells (left panel) versus non-permissive normal human cells (right panel). In permissive cells (e.g. tumor cells) PDK1-S135 phosphorylation stimulates parvovirus propagation and prolongs survival independently of growth factor signaling. In non-permissive normal human cells, due the failure of PVs to induce PDK1 stimulation, NS1 remains inactive.

Prolonging host cell survival under stress seems to be a common strategy used by viruses, to ensure sufficient progeny particle production and spread [24,28], and by cancer cells, for rapid proliferation and dissemination from the primary tissue [26,27]. It can be achieved through activation of the PDK/PKB signaling cascade. Here we reveal a new mechanism of PDK1/PKC/PKB upregulation, induced by PVs in permissive cells. After initial activation of PKCη by PDK1, PVs accelerate a PDK1-stimulating loop-back mechanism that depends on PKCη but not on growth factor signaling. As illustrated in Fig. 8 (left panel), this is achieved by enabling the ERM-family protein radixin to form with PKCη a complex mediating PDK1 phosphorylation at residue S135 (of human PDK1) or S138 (of mouse PDK1). Besides supporting activation of the short-lived PKCη necessary for NS1 replicative functions and cytotoxicity, this stimulation of PDK1 activity contributes to signaling through other downstream kinases, including PKB. Possibly in conjunction with other PV-triggered processes, this loop-back activation promoting cell metabolism and survival appears to extend the window of cell competence for sustaining PV replication by preventing premature death of infected cells.

As both PVs and cancer cells depend on activated PDK1 signaling and as host cells can overcome limitations to PV replication through malignant transformation [29], we suspected that the newly discovered mechanism might be active in human cancer cells and contribute to the natural oncotropism of PVs. We accordingly found PKCη/Rdx-mediated activation of PDK1/PKB signaling to occur in several PV-permissive human tumor cell lines, and this should be relevant to both tumor cell physiology and PV oncoselectivity (Fig. 8, compare the left and right panels).

Abnormal activation of the growth-factor-stimulated PI3K/PDK1/PKB signaling cascade is a common feature in cancer [9]. PDK1 is a crucial component thereof, controlling numerous downstream protein kinases, including PKB/Akt, SGK, S6K, RSK, and PKC isoforms. Thus, despite its universal role in cell homeostasis, accumulating evidence points to PDK1 as a valid target for cancer therapy. For example, PDK1 downregulation can inhibit migration and experimental metastasis of human breast cancer cells [30]. PDK1 depends strongly on extracellular stimuli. It is typically upregulated by phosphatidylinositols, whose production is driven by PI_3_K under the control of growth-factor-triggered signaling through receptor tyrosine kinases. Uncontrolled growth and dissemination of tumor cells require mechanisms activating this master kinase independently of the extracellular environment provided by the surrounding tissue. As previous studies show, this can be achieved through post-translational modifications triggering conformational changes [23,27]. The results presented here suggest a similar mode of PDK1 activation though PKCη/Rdx-driven phosphorylation of mouse PDK1:S138 (human PDK1:S135). The demonstration of PDK1phosphoS135 in some highly invasive cancers (e.g. malignant gliomas) leads us to advocate including PDK1phosphoS135 in the panel of tumor markers, as tumor cells acquiring this intracellular activation mechanism are likely to gain a significant growth advantage under conditions of restricted external stimuli.

We here identify PKCη as a major driver of PDK1 phosphorylation. Although somatic alterations of PKC genes seem rare in tumor cells, this kinase family has been implicated in cancer progression. PKC accumulation and activation have been found to correlate with both acquired resistance and poor prognosis in a number of cancers [16]. This has been attributed to the anti-apoptotic effects of PKCs and their ability to promote proliferation, anchorage-independent growth, and metastasis. Yet PKC-targeting cancer therapies have been hampered by the by the versatility of PKCs and the low isoform specificity of the inhibitors used, and the involvement of individual PKCs in cancer progression remains controversial. PKCη is thus reported to activate PKB/Akt1 (and thereby promote cell survival) in some cancers [31], but to down regulate it in others [32]. This discrepancy may be due to interaction of PKCη with different adaptor proteins. Here we identify the ERM-family protein radixin as an accessory protein controlling the activity and substrate specificity of PKCη. It is noteworthy that ERM proteins, like PKCs, have been implicated in cancer development, promoting growth and migration [21]. Because they interact with and regulate PKCs, they are likely to modulate the impact of these kinases on cancer development. Our present finding that the PKCη/Rdx complex acts as an intracellular regulator of PDK1 activity through S135 phosphorylation supports the view that ERM proteins can play a role in promoting carcinogenesis.

Altogether, our results suggest that PKCη/Rdx complex formation and concomitant PDK1phosphorylation represent a crucial step in both PV propagation and cancer progression. In MVM-infected A9 cells this event coincides with virus-induced translocation of the three proteins to a distinct microcompartment of the cell, i.e the perinuclear area. These data lead us to propose that the direct interaction of Rdx with PKCη modulates the substrate specificity of this kinase, enabling PKCη to target PDK1 and phosphorylate it at S138. Rdx compoexing with PKCη may depend on the previously reported Rdx-binding to the NS1“targeting”-domain (aa 278–379), although the ensuing Rdx phosphorylation by the NS1/CKIIα-complex does not appear to be required for PKCη/Rdx driven phosphorylation of PDK1 [22]. Upon interaction with NS1, Rdx may be translocated to the perinuclear area, where it was shown to be involved, together with NS1, in the formation and trafficking of exocytic vesicles [33]. Interestingly, MVM-infected A9 cells are characterized by the accumulation of cytoskeletal structures (e.g. tropomyosin filaments) around the nuclear lamina [34–36]. It is tempting to speculate that these perinuclear structures serve as scaffolds bringing together NS1, Rdx, PKCη, and PDK1, and organizing the sequential interactions of these proteins. The complexity of these interactions with multiple different cellular factors might account for the incapability of rodent PVs to induce this signaling in normal human cells.

H-1PV is currently being validated as a therapeutic agent in a phaseI/IIa clinical trial inpatients suffering from glioblastoma multiforme [2]. *In vitro*, these highly aggressive cancers prove extremely sensitive to H-1PV-induced killing, even when they have acquired resistance to common death inducers such as cisplatin or TRAIL [25]. The present study provides a first molecular clue to (H-1)PV oncoselectivity: on the one hand, growth-factor- and PI_3_K-independent activation of PDK1/(PKB) signaling may contribute to cancer progression in an unfavorable environment; on the other hand, the newly identified PKCη/Rdx-driven loop-back activation of PDK1 induced by PVs in cancer cells, but not in normal cells, appears to favor PV amplification and cytotoxicity and to counteract virus-induced stress responses. We thus propose PDK1phosphoS135 as a potential marker of both cancer progression and tumor sensitivity to parvovirotherapy.

## Material and Methods

### Ethics statement

Written informed consent was obtained from all patients. The study was approved by the local Ethics committee of the Medical Faculty of the University of Heidelberg, Germany (74–2000).

### Reagents

#### Primary antibodies

Antibodies against PKCη (C-15), Rdx (C-15), PDK1 (E-3), and PKB (C-20) were from Santa Cruz, antibodies against α-tubulin (B-5-1-2), PDK1 (#06-906), the Myc- (M5546) and Flag- (M2)-tags from Sigma, antibodies against actin (C-4) from MP Chemicals, antibodies against PDK1:P241 (ab32800) and PKCη P655 (ab5798) from Abcam, and antibodies against PKB-P308, from Upstate Biochemicals (#06-6701) or Geneway (#20-203420759). Rabbit antisera recognizing viral components have been described [33]. Radixin P564 phosphospecific antibodies were kindly provided by Prof. Dr. S. Tsukita [22]. Anti-PDK1:P138 antisera (#39, #40, #769) were generated by immunizing three different rabbits with the modified peptide TRERDVM-S(PO3H2)-RLDHP. Antisera were tested by ELISA for specific reactivity towards modified and non-modified peptides prior to immunoaffinity purification (Eurogentec). PDK1phosphoS138-specific mouse monoclonal antibodies were prepared using the same modified peptide.

#### Secondary antibodies

Horseradish-peroxidase-conjugated anti-rabbit and anti-mouse IgGs were from Promega, anti-goat and anti-sheep IgGs from Santa Cruz, anti-rat IgGs from Invitrogen. All fluorescent-dye-labeled IgGs were from Invitrogen.

#### Reagents

DAPI, propidium iodide (PI), and wortmannin (KY12420) were from Sigma, Mitotracker from Invitrogen, protein G Sepharose beads from MP, recombinant purified PKCη from Sigma and US Biologicals. Recombinant His-tagged Rdx was produced in HeLa cells by recombinant vaccinia virus expression and purified by affinity chromatography on Ni-NTA agarose columns [22,37].

### Effector constructs

#### Protein kinases

Myc-tagged wild-type PDK1 and dominant-negative (dn) PDK1:K204M [34], dnPDK1:S244A, and active PDK1*dl*PH [8], Flag-tagged PKCλ [34], Myc-tagged PKCη, flag-tagged constitutively active (ca) PKCηA160E, flag-tagged dnPKCηT512A [6,8], and flag-tagged dnCKIIαE81A [35,36] have been described.

#### ERM-family proteins

Flag-tagged dnEzT566A, caEzT566E, dnRdxT564A, caRdxT564E, dnRdx*dl*P, dnMoeT547A, and caMoeT547E have been described [22].

### Cells and viruses

#### Cell lines

All cell lines were maintained as monolayers in Dulbecco’s Modified Eagle’s Medium (DMEM) containing 10% fetal calf serum (FCS).

#### Viruses

MVMp (MVM) was propagated in A9 cells; hgH-1PV (H-1PV) is a variant, selected for propagation in human glioma cells [38]. It was amplified in NB324K cells. Stocks of full virions and empty capsids were obtained after CsCl density gradient purification and quantified by plaque assays [8]. *Recombinant adeno-associated viruses (rAAVs)*. rAAVs expressing effector constructs were generated in 293T cells in the absence of Ad5, by co-transfection with pAAV-P4-X (a plasmid containing AAV2-ITRs flanking the expression cassette) and pDG (a plasmid expressing helper functions) and titrated by dot blot hybridization [33].

### Site-directed mutagenesis and cloning procedures

Site-directed mutagenesis was performed by single or chimeric PCR [6]. PCRs were performed with an N-terminal primer (consisting of a unique restriction site generating blunt ends, followed by the Flag-tag or Myc-tag sequence plus 40 nts of effector-protein-coding sequence) and a C-terminal reverse primer (consisting of a unique XbaI or NotI site plus >30 nts from the coding sequence of interest). PCR fragments were cloned into pCR2.1 vectors (Invitrogen) and verified by sequencing.

#### Production of expression constructs for generating stably transfected cell lines

MVM NS1-inducible expression vectors were constructed from plasmid pP38 [6]. Wild-type and mutant Myc/Flag-tagged protein variants were excised from pCR2.1 vectors and ligated into similarly cleaved pP38 vector.

#### Expression constructs for bacterial production of recombinant proteins

The sequences encoding the N-terminal domains of wild-type and mutant PDK1 and the C-terminal domain of NS1 (fused to GFP) were generated by PCR, using a N-terminal primer bearing a SmaI restriction site for cloning the coding sequence in frame with the translation start of the bacterial expression plasmid so that the SmaI- and HindIII-cleaved PDK1x and NS1-C sequences could be cloned into Sma- and HindIII-cleaved pQE-32 (Qiagen).

#### rAAV-P4-X constructs

Effector genes were inserted into the Eco47III- and NotI-cleaved pAAV-P4-GFP vector [33] generating the corresponding pAAV-P4-X constructs.

### Generation of stably transfected A9 cell lines

Stable transfectants were generated with pP38-X and the selection plasmid pSV2neo or pTK-hyg at the molar ratio 25:1 [6]. Colonies were pooled after growth under selection and frozen stocks prepared. Experiments were performed in the absence of the drugs. Transfectants were kept in culture for less than 25 passages.

### Immunofluorescence microscopy and viability assays

Cells were grown on spot slides (Roth), mock-treated, rAAV-transfected, and/or PV-infected, and further incubated for the appropriate time. If applicable, wortmannin (0.5 μM) was added for 4 h at 37°C. Cell metabolism was measured by incubating live cells with Mitotracker (200 nM) for 30 min at 37°C. Necrosis was determined by incubating live cells with propidium iodide (1 μg/ml) for 30 min at 37°C. Cultures were fixed with 3% paraformaldehyde and permeabilized with 0.2% Triton X-100. Specimens were pre-adsorbed, incubated with primary antibodies, and stained with Alexa Fluor 488, 564, or 647-conjugated anti-species antibodies. DAPI (10 μg/ml) was added to the secondary antibody solutions. Analyses were performed with a Leica DMIRBE microscope and Powerscan software or with an Olympus Fluoview FV1000 confocal microscope for visualization of individual slices of a stack and quantified with ImageJ software [38].

#### Statistic evaluations

Data are expressed as mean values with standard deviation bars. The significance of changes due to the respective treatments were validated by student’s test at p-values p<0,01 and p<0,02, respectively.

### Co-immunoprecipitation assays

Cell extracts were prepared by incubating cell pellets for 30 min on ice in extraction buffer containing 20 mM Hepes-KOH, pH 7.5, 150 mM NaCl, 1 mM EDTA, 0.2% NP-40 and clarified by centrifugation. Supernatants were pre-adsorbed with FCS and protein G Sepharose for 2 h at room temperature before obtaining MycPKCη immunoprecipitates with anti-Myc overnight at 4°C. After washes in CoIp buffer, samples were analyzed by western blotting with rabbit anti-Myc.

### Western blot analysis

Cell extracts were produced by incubating cell pellets in extraction buffer containing 20 mM Hepes-KOH pH 7.5, 300 mM NaCl, 1 mM EDTA, 0.2% NP-40 on ice, and clarified by centrifugation. Tumor samples were processed for 40 sec in Lysing Matrix D (MP chemicals) with a Precellys24 homogenizer and clarified by centrifugation. Proteins were analyzed by SDS-PAGE, blotted onto nitrocellulose membranes, and identified with appropriate primary antibodies in 10% dry milk/PBS or 2% casein (phosphospecific antibodies), stained with horseradish-peroxidase-conjugated secondary antibodies for 1 h, and detected by chemiluminescence (Amersham) [33].

### Metabolic labeling, phosphorylated protein purification, and phosphopeptide analysis

Metabolic labeling and tryptic phosphopeptide analyses were performed as described [39]. Cultures were infected with MVM (30 pfu/cell), incubated for 24 h, and labeled for 4 h in medium containing 0.1 nCi/cell of [^32^P] orthophosphate (MP biochemicals). Labeled proteins were isolated by immunoprecipitation, purified by SDS-PAGE, and blotted onto PVDF membranes. ^32^P-labeled proteins were revealed by autoradiography, excised, and the membrane-bound proteins were digested with 50 units of trypsin. Two-dimensional tryptic phosphopeptide analysis was performed on thin-layer cellulose plates (Merck) by electrophoresis in pH 1.9 buffer followed by chromatography in phosphochromatography buffer.

### *In vitro* kinase reactions

*In vitro* kinase reactions were performed with recombinant PKCη (supplemented or not with HisRdx) together with 100 ng bacterially expressed NS1_C_ or wild-type or mutant PDK1_N446_ as substrate. Assays were performed for 40 min at 37°C with γ[^32^P]ATP (30 μCi) in 50 μl labeling buffer, in the presence of TPA and PS. Reaction products were purified by SDS-PAGE and further processed for tryptic phosphopeptide analysis [39].

## Supporting Information

S1 Fig Impact of PKCη and radixin on endogenous PDK1 activation in MVM-infected A9 cells. A9 cells and derivatives producing a dominant-negative (dn) variant of casein kinase II (dnCKIIαE81A) [35,36], PKCη (dnPKCηT512A) [6], or radixin (dnRdx*dl*[P]) [22] or a constitutively active variant of PKCη (caPKCηA160E) [6] or radixin (caRdxT564E) [22] under the control of the PV-inducible P38-promoter were infected (or not) with MVM and analyzed at the indicated times p. i. with antisera specifically recognizing active (autophosphorylated) PDK1phosphoS244 (PDK:P244). The total amount of PDK1 was determined in parallel. 14-3-3 family proteins were used as internal loading control.
(PPT)

S2 Fig Cell permissiveness for H-1PV infection. Normal diploid human foreskin fibroblasts (BJ-1), embryonic lung fibroblasts (MRC-5), and human glioblastoma-derived cell lines (NCH149 and NCH82) were infected (or not) at 30 pfu/cell with H-1PV and analyzed for viral DNA amplification and NS1 synthesis at the indicated times p.i. (A) Total DNA was extracted from harvested cells and analyzed by Southern blotting for its content in viral replicative-form DNA (mRF, dRF) and single-stranded virion DNA (ssDNA). (B) NS1 was detected and necrosis measured 36 h after H-1PV infection. For NS1 detection, cells were fixed with paraformaldehyde and analyzed by indirect fluorescence microscopy with NS1-specific SP8 antiserum. The proportion of necrotic cells was determined by propidium iodide incorporation for 30 min.
(PPTX)

S3 Fig Impact of PKCη and Rdx on cell metabolic activity and survival. The indicated cell lines were transduced with a rAAV (10^4^ viral genomes/cell) expressing a dominant-negative (dn) or constitutively active (ca) form of the indicated signaling protein under control of the PV P4 promoter. 72 h post transduction, the cells were labeled for 30 min with Mitotracker and mitochondrial activity was measured by confocal laser scanning microscopy, quantified with Image J software as relative light intensity per cell. In parallel, proportions of dead cells and apoptotic cells were measured, respectively, by PI (necrosis) and DAPI staining (detection of apoptotic bodies). (A) Immunofluorescences of representative samples of rAAV-treated A9 cells. (B) Summarized data from rAAV-treated PDK1phosphoS135 positive NCH82 and -negative BJ-1 cells, expressed as a percentage of the value obtained for mock-treated cells. The data presented are means with standard-deviation bars of three individual experiments, each involving > 200 cells per sample. Statistical significant changes (p<0,01) due to the treatment are marked by astericks and highlighted in black. dnPDK, PDK1K204M; dnPKCη, PKCηT512A; caPKCη, PKCηA160E; dnRdxA, RdxT564A; caRdxE, RdxT564E; dnRdxP, Rdx*dl*[P]; for comparison, viability was also measured 24 h after infection with H-1PV. rAAV-mediated transduction efficiencies were checked by confocal microscopy (S7 Fig.).
(PPT)

S4 Fig Impact of mutations at candidate PKC phosphorylation sites on PDK1 activity: replacement of putative target serine/threonine residues with glutamic/aspartic acid. A9 cells grown on spot slides were transfected with plasmids expressing the indicated PDK1 mutants. 48 h post-transfection, PDK1 auto- and *trans-*phosphorylation activities were tested by cell immunostaining for PDK1phosphoS244 or PKB:phosphoT308 and examination by confocal laser scanning microscopy. Mock- and MVM-infected A9 cells were used, respectively, as negative and positive controls. Scale bar, 8 μM.
(PPT)

S5 Fig Impact of caPDK1 on the growth factor dependence of cell metabolic activity and survival. Each indicated cell line was transduced with a rAAV (10^4^ rAAV genomes/cell) expressing mutant PDK1 under the control of the PV P4 promoter. 72 h post transduction, the cells were treated (or not) for 4 h with 0.5 μM wortmannin prior to labeling for 30 min with Mitotracker. Mitochondrial activity and cell death were measured as described in the legend of Figs. 4 and S3. The constitutively active mutant PDK1:S138E expressed in BJ-1 cells significantly (p<0,01) reconstituted metabolic activity and prevented cells undergoing death through necrosis. Thus, PDK1:S138E appeared to render cell viability independent of growth factor signaling via the PI3 kinase (marked black). It should be noted that PDK1:S138E mimics PDK1phophoS135 modification detected in non-transduced NCH82 cells (Fig. 4A). Transduction efficiencies were checked by confocal microscopy (S7 Fig.).
(PPT)

S6 Fig Detection of PDK1phosphoS135 in human glioma tissues. Cryosections of human brain tumors (T) were analyzed for the presence of PDK1phosphoS135 by immunostaining with phosphospecific monoclonal antibodies (PDKp135) and counterstaining with DAPI. Muscle tissue samples (Mu) were used as negative controls. Scale bar, 50 μm.
(PPTX)

S7 Fig Transfection of target cell lines with rAAVs expressing effector genes under the control of the PV P4 promoter. A9, MRC-5, BJ-1, NCH149, and NCH82 cells grown on spot slides were transfected with rAAV-P4-X (10^4^ genomes/cell). 72 h post-transfection, the cells were fixed with paraformaldehyde and analyzed for the presence of recombinant proteins by immunofluorescence confocal laser scanning microscopy, with antibodies recognizing GST (rAAV) or the fused N-terminal epitope Myc (dnPDK; S138E, S138E, S237D, S237E, S265E, T516E, T525E) or Flag (dnPKCη, caPKCη, RdxA, RdxE, RdxP, RdxY). Scale bar, 30 μm.
(PPT)
