# Supplementary figures and images for: PKCη/Rdx-driven Phosphorylation of PDK1: A Novel Mechanism Promoting Cancer Cell Survival and Permissiveness for Parvovirus-induced Lysis
Source: PLoS Pathog. 2015 Mar 5;11(3):e1004703. doi: 10.1371/journal.ppat.1004703 (PMC4351090; doi:10.1371/journal.ppat.1004703)

## Slide 1
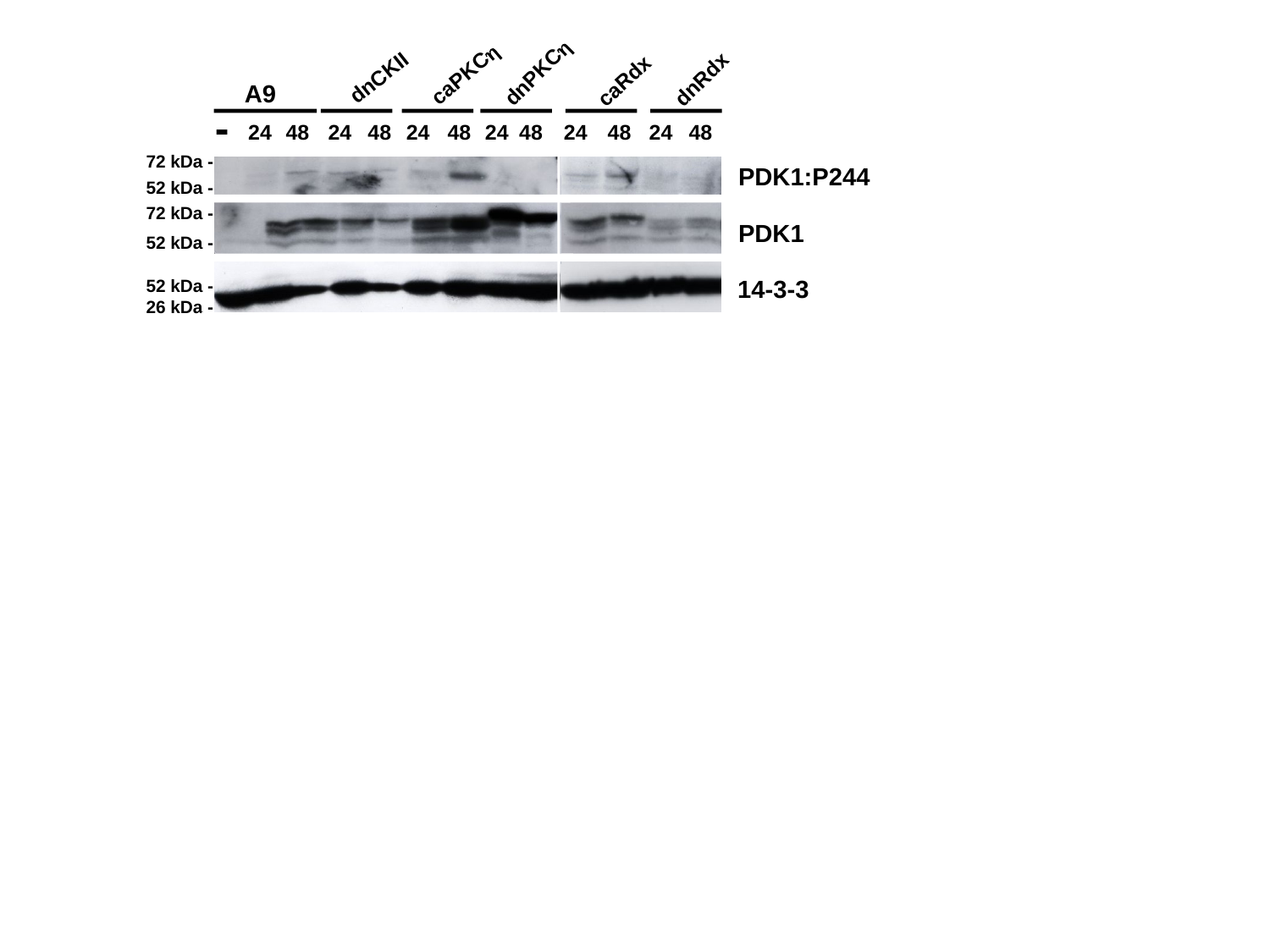

dnPKCη
caPKCη
dnCKII
dnRdx
caRdx
A9
-
24
48
24
48
24
48
24
48
24
48
24
48
72 kDa -
52 kDa -
72 kDa -
52 kDa -
52 kDa -
26 kDa -
PDK1:P244
PDK1
14-3-3

Supplement: S1 Fig — A9 cells and derivatives producing a dominant-negative (dn) variant of casein kinase II (dnCKIIαE81A) [35,36], PKCη (dnPKCηT512A) [6], or radixin (dnRdxdl[P]) [22] or a constitutively active variant of PKCη (caPKCηA160E) [6] or radixin (caRdxT564E) [22] under the control of the PV-inducible P38-promoter were infected (or not) with MVM and analyzed at the indicated times p. i. with antisera specifically recognizing active (autophosphorylated) PDK1phosphoS244 (PDK:P244). The total amount of PDK1 was determined in parallel. 14-3-3 family proteins were used as internal loading control. (PPT) [file ppat.1004703.s001.ppt]

## Slide 1
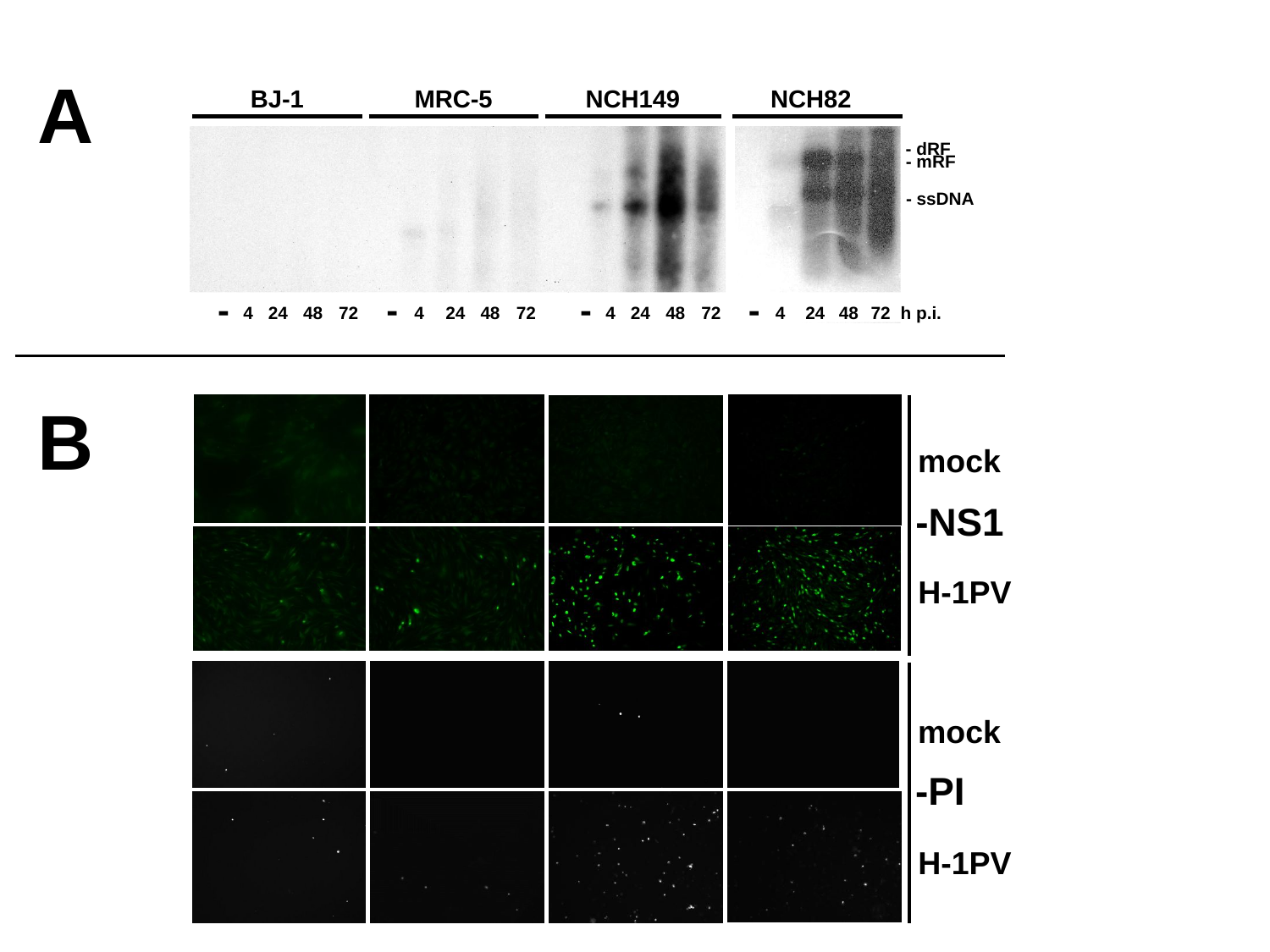

A
BJ-1
MRC-5
NCH149
NCH82
- dRF
- mRF
- ssDNA
-
4
24
48
72
-
-
4
24
48
72
-
4
24
48
72
4
24
48
72
h p.i.
B
mock
-NS1
H-1PV
mock
-PI
H-1PV

Supplement: S2 Fig — Normal diploid human foreskin fibroblasts (BJ-1), embryonic lung fibroblasts (MRC-5), and human glioblastoma-derived cell lines (NCH149 and NCH82) were infected (or not) at 30 pfu/cell with H-1PV and analyzed for viral DNA amplification and NS1 synthesis at the indicated times p.i. (A) Total DNA was extracted from harvested cells and analyzed by Southern blotting for its content in viral replicative-form DNA (mRF, dRF) and single-stranded virion DNA (ssDNA). (B) NS1 was detected and necrosis measured 36 h after H-1PV infection. For NS1 detection, cells were fixed with paraformaldehyde and analyzed by indirect fluorescence microscopy with NS1-specific SP8 antiserum. The proportion of necrotic cells was determined by propidium iodide incorporation for 30 min. (PPTX) [file ppat.1004703.s002.pptx]
