# Supplementary material for: PKCη/Rdx-driven Phosphorylation of PDK1: A Novel Mechanism Promoting Cancer Cell Survival and Permissiveness for Parvovirus-induced Lysis
Source: PLoS Pathog. 2015 Mar 5;11(3):e1004703. doi: 10.1371/journal.ppat.1004703 (PMC4351090; doi:10.1371/journal.ppat.1004703)
Supplement: S3 Fig — The indicated cell lines were transduced with a rAAV (104 viral genomes/cell) expressing a dominant-negative (dn) or constitutively active (ca) form of the indicated signaling protein under control of the PV P4 promoter. 72 h post transduction, the cells were labeled for 30 min with Mitotracker and mitochondrial activity was measured by confocal laser scanning microscopy, quantified with Image J software as relative light intensity per cell. In parallel, proportions of dead cells and apoptotic cells were measured, respectively, by PI (necrosis) and DAPI staining (detection of apoptotic bodies). (A) Immunofluorescences of representative samples of rAAV-treated A9 cells. (B) Summarized data from rAAV-treated PDK1phosphoS135 positive NCH82 and -negative BJ-1 cells, expressed as a percentage of the value obtained for mock-treated cells. The data presented are means with standard-deviation bars of three individual experiments, each involving > 200 cells per sample. Statistical significant changes (p<0,01) due to the treatment are marked by astericks and highlighted in black. dnPDK, PDK1K204M; dnPKCη, PKCηT512A; caPKCη, PKCηA160E; dnRdxA, RdxT564A; caRdxE, RdxT564E; dnRdxP, Rdxdl[P]; for comparison, viability was also measured 24 h after infection with H-1PV. rAAV-mediated transduction efficiencies were checked by confocal microscopy (S7 Fig.). (PPT) [file ppat.1004703.s003.ppt]

## Slide 1
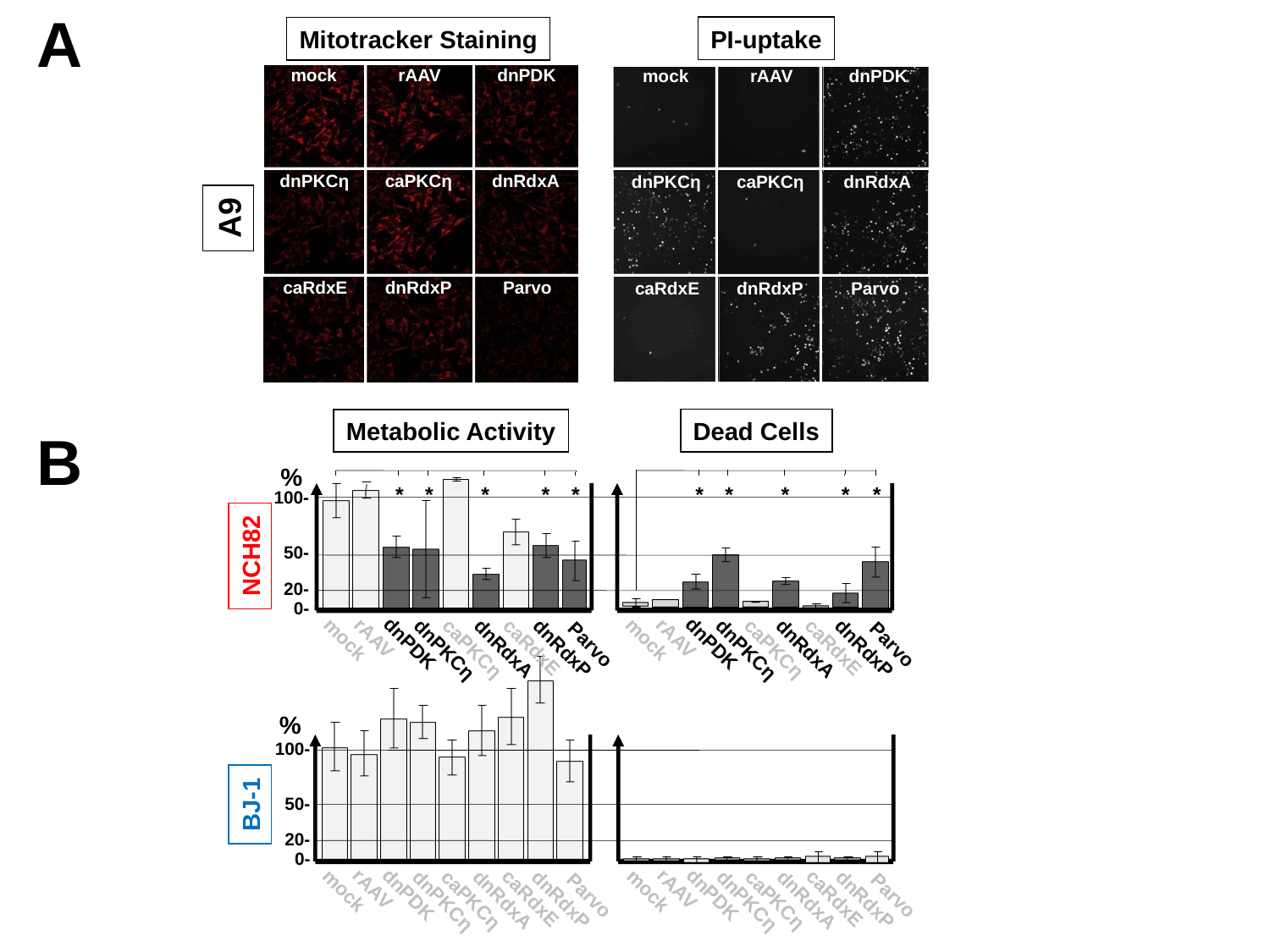

A
PI-uptake
Mitotracker Staining
mock
rAAV
dnPDK
mock
rAAV
dnPDK
dnPKCη
caPKCη
dnRdxA
dnPKCη
caPKCη
dnRdxA
caRdxE
dnRdxP
Parvo
caRdxE
dnRdxP
Parvo
A9
Dead Cells
Metabolic Activity
%
100-
50-
20-
0-
rAAV
mock
dnPDK
Parvo
caRdxE
dnRdxP
caPKCη
dnRdxA
dnPKCη
rAAV
mock
dnPDK
Parvo
caRdxE
dnRdxP
caPKCη
dnRdxA
dnPKCη
NCH82
%
100-
50-
20-
0-
BJ-1
rAAV
mock
dnPDK
Parvo
caRdxE
dnRdxP
caPKCη
dnRdxA
dnPKCη
rAAV
mock
dnPDK
Parvo
caRdxE
dnRdxP
caPKCη
dnRdxA
dnPKCη
B
*
*
*
*
*
*
*
*
*
*
