# Supplementary material for: PKCη/Rdx-driven Phosphorylation of PDK1: A Novel Mechanism Promoting Cancer Cell Survival and Permissiveness for Parvovirus-induced Lysis
Source: PLoS Pathog. 2015 Mar 5;11(3):e1004703. doi: 10.1371/journal.ppat.1004703 (PMC4351090; doi:10.1371/journal.ppat.1004703)
Supplement: S4 Fig — A9 cells grown on spot slides were transfected with plasmids expressing the indicated PDK1 mutants. 48 h post-transfection, PDK1 auto- and trans-phosphorylation activities were tested by cell immunostaining for PDK1phosphoS244 or PKB:phosphoT308 and examination by confocal laser scanning microscopy. Mock- and MVM-infected A9 cells were used, respectively, as negative and positive controls. Scale bar, 8 μM. (PPT) [file ppat.1004703.s004.ppt]

## Slide 1
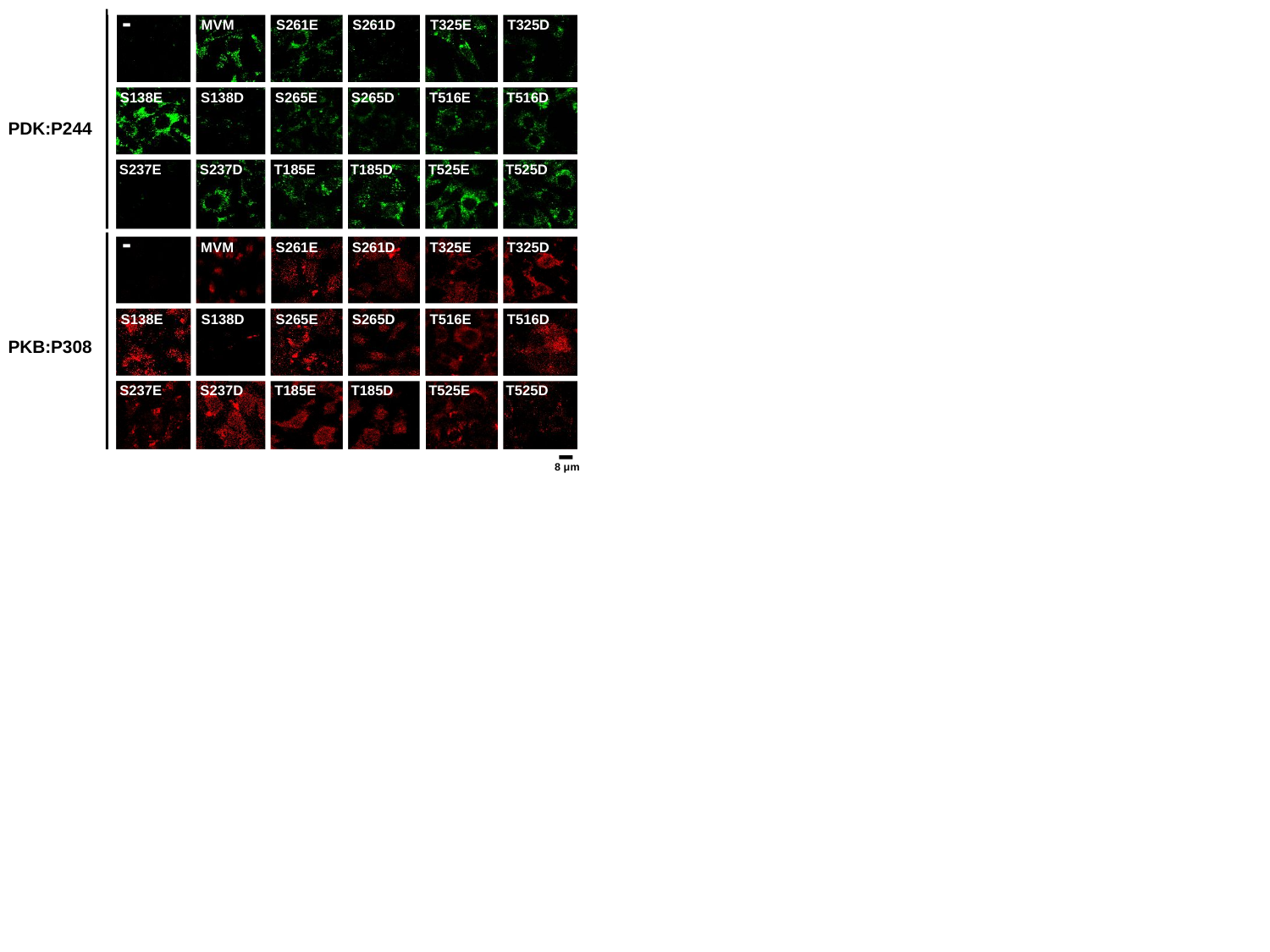

-
MVM
S261E
S261D
T325E
T325D
S138E
S138D
S265E
S265D
T516E
T516D
PDK:P244
S237E
S237D
T185E
T185D
T525E
T525D
-
MVM
S261E
S261D
T325E
T325D
S138E
S138D
S265E
S265D
T516E
T516D
PKB:P308
S237E
S237D
T185E
T185D
T525E
T525D
8 μm
