# Supplementary material for: PKCη/Rdx-driven Phosphorylation of PDK1: A Novel Mechanism Promoting Cancer Cell Survival and Permissiveness for Parvovirus-induced Lysis
Source: PLoS Pathog. 2015 Mar 5;11(3):e1004703. doi: 10.1371/journal.ppat.1004703 (PMC4351090; doi:10.1371/journal.ppat.1004703)
Supplement: S5 Fig — Each indicated cell line was transduced with a rAAV (104 rAAV genomes/cell) expressing mutant PDK1 under the control of the PV P4 promoter. 72 h post transduction, the cells were treated (or not) for 4 h with 0.5 μM wortmannin prior to labeling for 30 min with Mitotracker. Mitochondrial activity and cell death were measured as described in the legend of Figs. 4 and S3. The constitutively active mutant PDK1:S138E expressed in BJ-1 cells significantly (p<0,01) reconstituted metabolic activity and prevented cells undergoing death through necrosis. Thus, PDK1:S138E appeared to render cell viability independent of growth factor signaling via the PI3 kinase (marked black). It should be noted that PDK1:S138E mimics PDK1phophoS135 modification detected in non-transduced NCH82 cells (Fig. 4A). Transduction efficiencies were checked by confocal microscopy (S7 Fig.). (PPT) [file ppat.1004703.s005.ppt]

## Slide 1
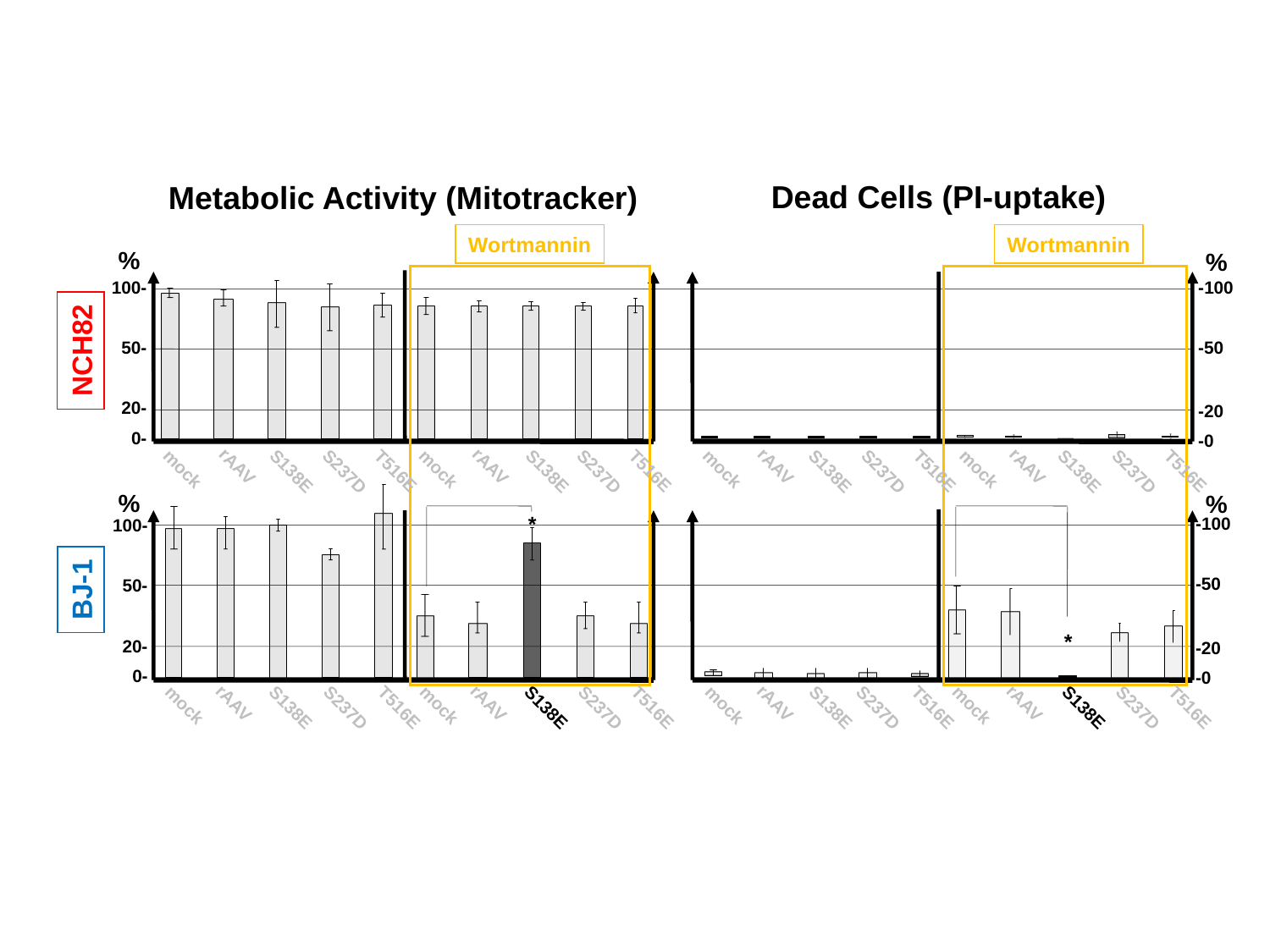

Dead Cells (PI-uptake)
Metabolic Activity (Mitotracker)
Wortmannin
Wortmannin
%
%
100-
50-
20-
0-
-100
-50
-20
-0
NCH82
rAAV
mock
T516E
S138E
S237D
rAAV
mock
T516E
S138E
S237D
rAAV
mock
T516E
S138E
S237D
rAAV
mock
T516E
S138E
S237D
%
%
-100
-50
-20
-0
100-
50-
20-
0-
BJ-1
rAAV
mock
T516E
S138E
S237D
rAAV
mock
T516E
S138E
S237D
rAAV
mock
T516E
S138E
S237D
rAAV
mock
T516E
S138E
S237D
*
*
