# Supplementary material for: PKCη/Rdx-driven Phosphorylation of PDK1: A Novel Mechanism Promoting Cancer Cell Survival and Permissiveness for Parvovirus-induced Lysis
Source: PLoS Pathog. 2015 Mar 5;11(3):e1004703. doi: 10.1371/journal.ppat.1004703 (PMC4351090; doi:10.1371/journal.ppat.1004703)
Supplement: S6 Fig — Cryosections of human brain tumors (T) were analyzed for the presence of PDK1phosphoS135 by immunostaining with phosphospecific monoclonal antibodies (PDKp135) and counterstaining with DAPI. Muscle tissue samples (Mu) were used as negative controls. Scale bar, 50 μm. (PPTX) [file ppat.1004703.s006.pptx]

## Slide 1
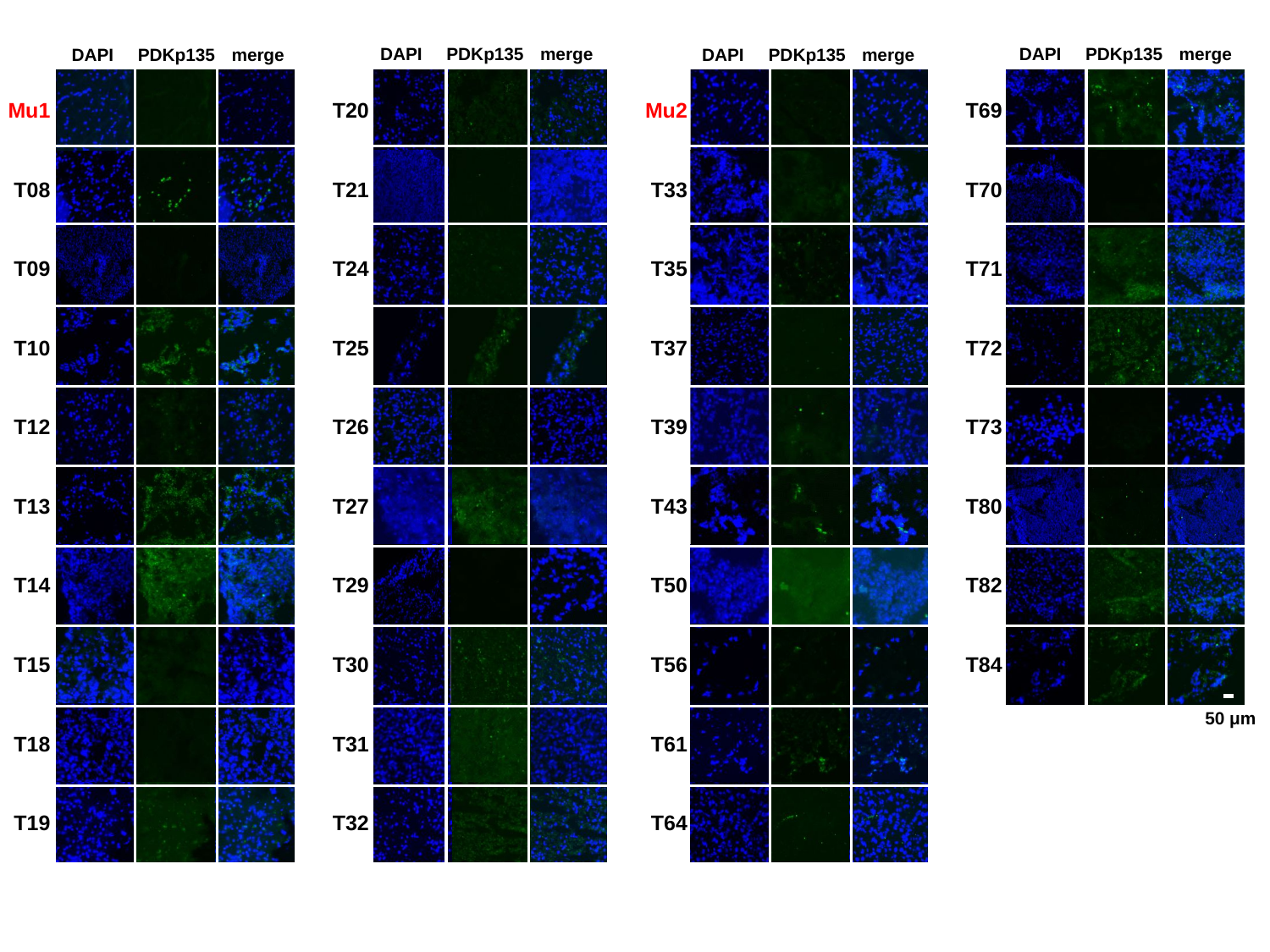

DAPI
PDKp135
merge
T20
T21
T24
T25
T26
T27
T29
T30
T31
T32
DAPI
PDKp135
merge
T69
T70
T71
T72
T73
T80
T82
T84
50 μm
DAPI
PDKp135
merge
Mu2
T33
T35
T37
T39
T43
T50
T56
T61
T64
DAPI
PDKp135
merge
Mu1
T08
T09
T10
T12
T13
T14
T15
T18
T19
