# Supplementary material for: PKCη/Rdx-driven Phosphorylation of PDK1: A Novel Mechanism Promoting Cancer Cell Survival and Permissiveness for Parvovirus-induced Lysis
Source: PLoS Pathog. 2015 Mar 5;11(3):e1004703. doi: 10.1371/journal.ppat.1004703 (PMC4351090; doi:10.1371/journal.ppat.1004703)
Supplement: S7 Fig — A9, MRC-5, BJ-1, NCH149, and NCH82 cells grown on spot slides were transfected with rAAV-P4-X (104 genomes/cell). 72 h post-transfection, the cells were fixed with paraformaldehyde and analyzed for the presence of recombinant proteins by immunofluorescence confocal laser scanning microscopy, with antibodies recognizing GST (rAAV) or the fused N-terminal epitope Myc (dnPDK; S138E, S138E, S237D, S237E, S265E, T516E, T525E) or Flag (dnPKCη, caPKCη, RdxA, RdxE, RdxP, RdxY). Scale bar, 30 μm. (PPT) [file ppat.1004703.s007.ppt]

## Slide 1
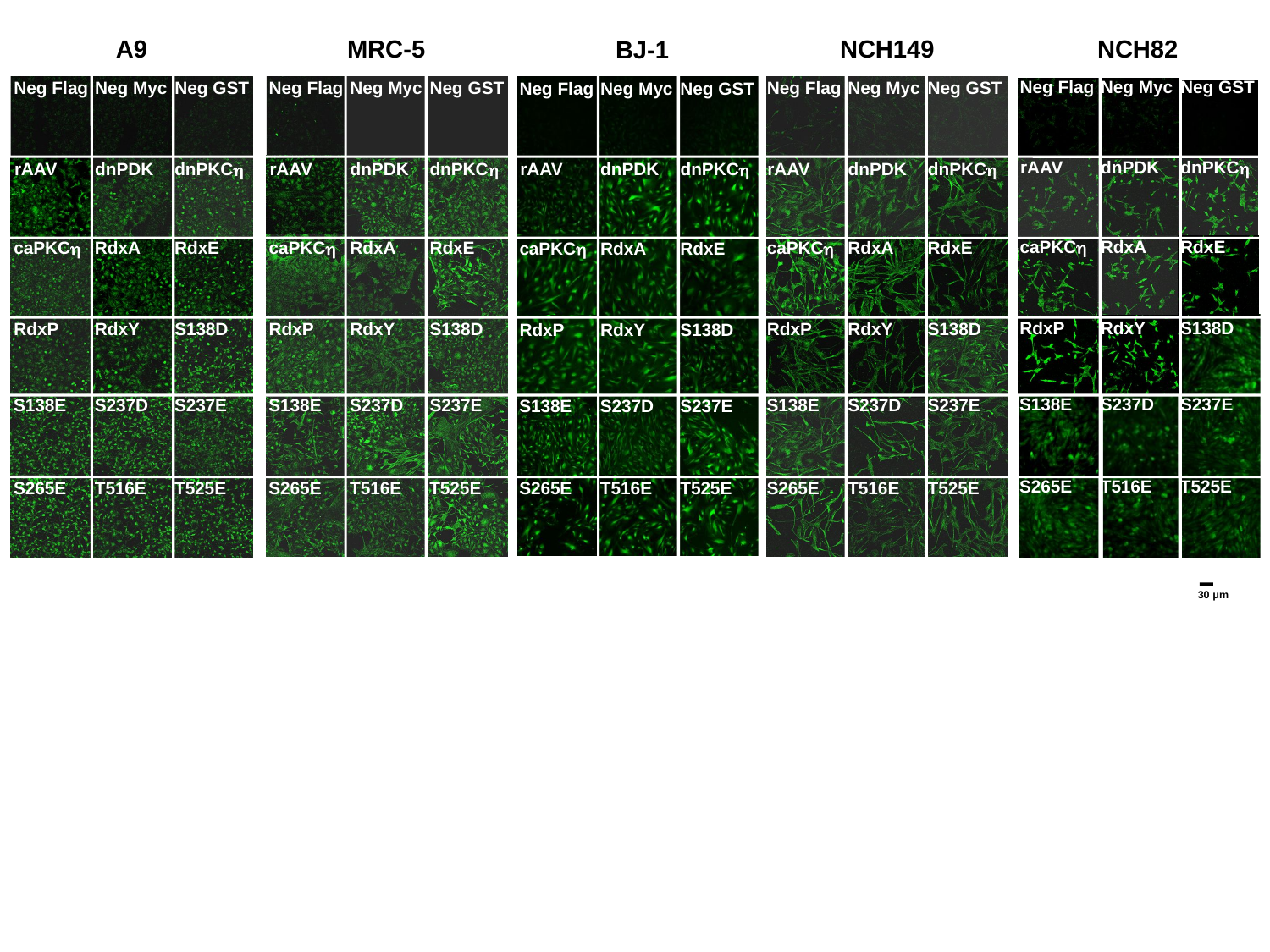

A9
MRC-5
NCH149
NCH82
BJ-1
Neg Flag
Neg Myc
Neg GST
rAAV
dnPDK
dnPKCη
caPKCη
RdxA
RdxE
RdxP
RdxY
S138D
S138E
S237D
S237E
T525E
S265E
T516E
Neg Flag
Neg Myc
Neg GST
rAAV
dnPDK
dnPKCη
caPKCη
RdxA
RdxE
RdxP
RdxY
S138D
S138E
S237D
S237E
S265E
T516E
T525E
Neg Flag
Neg Myc
Neg GST
rAAV
dnPDK
dnPKCη
caPKCη
RdxA
RdxE
RdxP
RdxY
S138D
S138E
S237D
S237E
S265E
T516E
T525E
Neg Flag
Neg Myc
Neg GST
rAAV
dnPDK
dnPKCη
caPKCη
RdxA
RdxE
RdxP
RdxY
S138D
S138E
S237D
S237E
S265E
T516E
T525E
Neg Flag
Neg Myc
Neg GST
rAAV
dnPDK
dnPKCη
caPKCη
RdxA
RdxE
RdxP
RdxY
S138D
S138E
S237D
S237E
S265E
T516E
T525E
30 μm
